# Top-performing girls are more impactful peer role models than boys, teachers say

**DOI:** 10.1073/pnas.2421436122

**Published:** 2025-02-06

**Authors:** Sofoklis Goulas, Rigissa Megalokonomou, Panagiotis Sotirakopoulos

**Affiliations:** ^a^Economic Studies Program, Brookings Institution, Washington, DC 20036; ^b^Department of Economics, Monash University, Melbourne, VIC 3145, Australia; ^c^Bankwest Curtin Economics Centre, Curtin University, Perth, WA 6102, Australia

**Keywords:** teacher gender stereotypes, randomized controlled trial, peer role models, STEM

## Abstract

We examine teachers’ perceptions toward top-performing students and their role model influence on others in an online survey-based experiment. We randomly expose teachers to profiles of top-performing students and inquire whether they consider the profiled top performers to be influential role models. These profiles varied by gender and field of study (STEM or Non-STEM). Our findings show that teachers perceive top-performing girls as more influential peer role models compared to top-performing boys ($\hat{\beta }=0.289$; P<0.001). We also investigate the qualities teachers perceive top performers who are successful role models to have. We show that teachers associate a greater sense of learning autonomy and sense of being an example with top-performing girls compared to top-performing boys. Estimated effects are more pronounced among teachers with children and teachers in urban areas. Administrative data from a representative sample show limited observed differences between top-performing boys’ and girls’ educational outcomes that could justify the differences in teachers’ gender perceptions. These findings carry significant implications for education, as teachers play a crucial role in the cultivation of positive externalities between students.

## Significance

We elicit teacher perceptions about top performers, who hold the potential of influencing the education production function of other students. If teachers consistently perceive some students, even top performers, as less impactful role models, teachers may limit the set or impact of available role models in the classroom, potentially limiting the positive externalities between students. Teachers are found to perceive top-performing girls as more impactful role models than top-performing boys in an online survey-based randomized experiment. Additionally, we explore the qualities linked to top performers’ role model status, finding that teachers perceive top-performing girls to possess an increased sense of learning autonomy and sense of being an example compared to top-performing boys.

Role models are exceedingly valuable in a person’s life (1, 2). They are usually exemplary individuals who inspire others to ideate a potential self and take steps to realize their potential (3–5). Standard classroom-based instruction, in particular, relies heavily on role model influences among peers. This is based on the “shining light” paradigm, which suggests that having exemplary students in the classroom has positive externalities for other students through imitation and motivation (6–8). To date, peer role models are believed to [1] increase the effectiveness of schooling, and [2] facilitate positive behavioral spillovers, amplifying education intervention effects (9, 10).

Teachers play a vital role in guiding and inspiring students to look up to certain peers as role models (11). They may, for instance, guide students to emulate the study habits of diligent classmates. As a result, role model influences in observational outcomes, such as test scores, may be confounded by teachers who may or may not facilitate peer role model influences in their classrooms. Whether teachers create a classroom culture that is conducive to role model interactions may depend on their own perceptions and stereotypes. For example, research has shown that teachers may have significant gender stereotypes (12–18). In this paper, we conduct a randomized survey-based experiment to elicit teacher perceptions regarding the role model influence of top-performing boys and girls. While teachers may recognize the ongoing discourse concerning the influence of role models, there remains a paucity of empirical evidence addressing their perceptions regarding the effects of these role models.

Top performers may be considered as role models for their peers for three reasons. First, top performers usually demonstrate a strong commitment to academic excellence (19). Thus, their achievements may inspire their peers, encouraging them to strive for similar success. Second, top-performing students may exhibit leadership qualities, such as confidence, autonomy, and responsibility in their approach to learning and collaboration (20). Third, exceptional students often display positive behavior and attitudes toward learning (21). Teachers and classmates may recognize these traits as role model qualities and as influential in molding a classroom culture conducive to learning.

Separating teachers’ perceptions about the role model potential of students and other student characteristics is challenging in nonexperimental settings. Top-performing boys and girls, for instance, may have different academic profiles, personalities, and other traits relevant to their role model influence on their peers. Our online survey-based experiment addresses this challenge by presenting fictitious top-performer profiles. These profiles are identical except for two manipulated characteristics: the gender of the top performer and type of subject they excel in (STEM- or Non-STEM-related).

Prior studies suggest that girls may benefit more from the presence of female role models than boys. Specifically, research has found positive and significant effects from female mentors on female students’ productivity (22–24), as well as the positive influence of female peers on female students (25–27). The literature has also shown that a higher share of high-achieving girls in the classroom improves girls’ test scores and the overall classroom learning productivity (28–30). Two key mechanisms have been proposed. First, girls may be less disruptive in the classroom than boys, creating an environment that may be more conducive to learning (30, 31). Second, girls may be higher-performing than boys, leading to positive ability spillover effects on others (32, 33).

In this study, we conducted a survey-based randomized controlled trial (RCT) among teachers in Greece to understand whether they view top-performing boys and girls as role models. Teachers were shown pictures of top-performing students writing on a whiteboard with varied gender and whiteboard content (STEM- or Non-STEM-related) to gather their perceptions. Our research design allows us to explore the intersection between perceived role model influences and gender stereotypes about STEM and Non-STEM fields. Specifically, we explore whether teachers perceive a top-performing girl in STEM as a more impactful role model than a top-performing boy in the same field (34). This inquiry arises from the prevailing notion that proficiency in STEM subjects is traditionally associated more with boys than with girls (35, 36).

Our first-order result is that teachers confirm that role model influences among classroom peers exist. We also show that teachers recognize top-performing girls as wielding a greater influence as peer role models compared to boys, particularly evident within Non-STEM subject areas. Our analysis shows the gendered nature of teacher perceptions regarding role models, impacting not only short-term outcomes like test scores but also shaping longer-term decisions such as track selection, college major preferences, and career trajectories.

We also explore the qualities underpinning perceived role model influences, examining whether top performers are perceived to have heightened confidence, learning autonomy, or a stronger sense of being an example for others (3, 4, 20, 25, 37, 38). Our findings reveal that teachers attribute a greater sense of autonomy and sense of being an example to top-performing girls compared to top-performing boys. These perceived attributes among top-performing girls may help explain why teachers perceive them as having a greater impact on their classmates.

We complement our RCT results with two empirical investigations using administrative data on test scores and attendance from 120 public high schools in Greece. The first one explores whether teachers’ beliefs reflect statistical bias related to gender differences in top performers’ academic performance. We find scant evidence of differences in scores or attendance of top-performing boys and girls, effectively dispelling the notion of substantial statistical bias among teachers (i.e., gender-based perceptions may not mirror actual gender gaps in top-performing students’ academic performance and conduct). In a second investigation, we explore the impact of top-performing boys and girls on their peers’ academic outcomes. We compare the end-of-year scores of students in quasi-randomly formed classrooms with a top-performing girl with scores of students in classrooms with a top-performing boy. We find that students quasi-randomly assigned (in the beginning of the year) in a classroom with a top-performing girl have a significantly higher end-of-year performance relative to students in classrooms with a top-performing boy. These findings corroborate our RCT results and substantiate teachers’ perceptions that top-performing girls may be more influential role models than top-performing boys.

We contribute to the existing literature in several important ways. First, we contribute to the literature on teacher gender stereotypes. We elicit teacher perceptions about top performers, who hold the potential of influencing the education production function of other students. Our experimental findings reveal an additional dimension of teachers’ bias that may affect their behaviors and attitudes. These perceptions can shape whether teachers actively guide other students’ attention toward these high achievers and may also reflect prevailing social norms. Diagnosing biases in social norms is a crucial first step in behavioral change (39). Second, we contribute to a growing literature on the role model function of school peers (25). Given the extensive time students spend in schools and the developmental flexibility inherent in school-age children, educational settings offer fertile ground for the cultivation of role model influences. Third, our study illuminates the qualities teachers perceive influential role models to have. These insights are valuable in crafting interventions aimed at harnessing and amplifying the positive impact of role models. For instance, interventions targeting the strengthening of learning autonomy may serve to propagate role model influences among peers.

## Current Study.

Drawing on prior education research (40–42), we designed a randomized online survey-based experiment to investigate teachers’ perceptions of the role model influence of top-performing boys and girls on their peers. Collaborating closely with the Ministry of Education, we randomly selected local school authorities of elementary and secondary education across Greece. Subsequently, school principals facilitated teacher participation in our online RCT, conducted via computer labs at schools in 2022. Participating teachers were provided with a link to the digital Qualtrics survey. To incentivize participation, we pledged a donation of 0.50 Euro to a philanthropic foundation of each participant’s choosing for every completed response. Spanning the entirety of the country geographically (as illustrated in *SI Appendix*, Fig. S5), our study was approved by the Institutional Review Board at Stanford University, with informed consent obtained from all participating teachers.

We collected data on teachers’ demographic characteristics, subject specializations, personal educational experiences, explicit biases, and perceived grading leniency. Specifically, we explored their personal educational journeys, including their ability to recall the gender of top and second-best performers in their own early high school classroom—effectively tracing potential cognitive imprints of role models from their formative years to discern any predispositions toward the role of top performers as influential models today. We captured explicit biases in two ways. First, we inquired whether teachers demonstrate leniency toward girls as opposed to boys. Second, we probed teachers regarding their perceptions of gender associations with particular occupations.

**Fig. 1.**
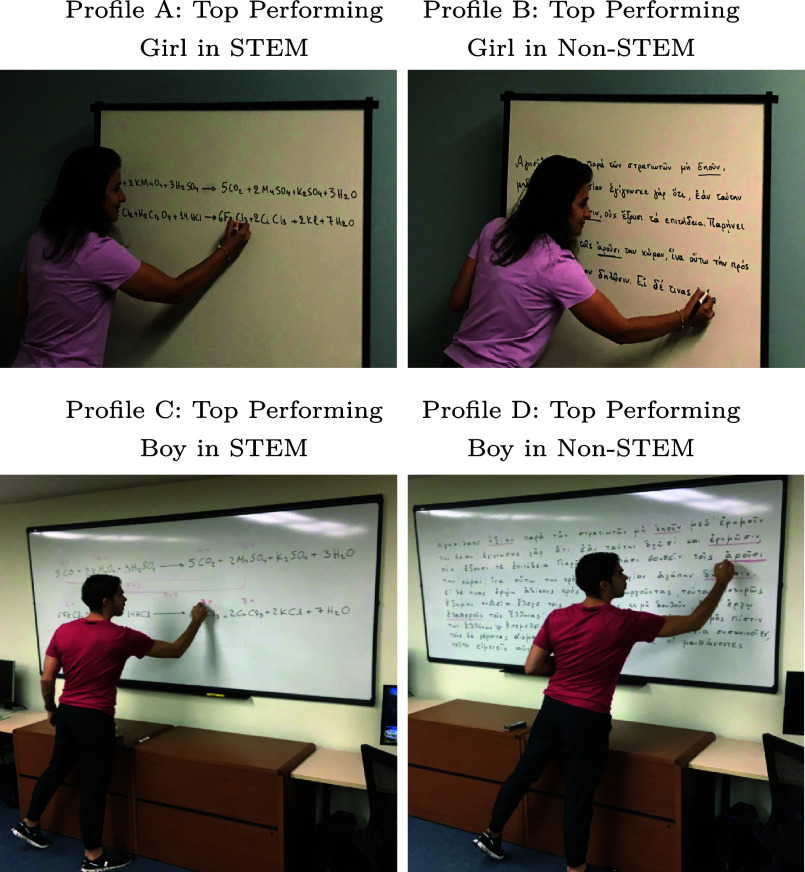
Randomized survey experiment profiles. Each participant was randomly exposed to one of the following treatment scenarios: top-performing girl in STEM (Fig. 1 Profile A), top-performing girl in Non-STEM (Fig. 1 Profile B), top-performing boy in STEM (Fig. 1 Profile C), and top-performing boy in Non-STEM (Fig. 1 Profile D).

Participants were then shown one of four profiles randomly selected: a top-performing girl in STEM, a top-performing girl in Non-STEM, a top-performing boy in STEM, and a top-performing boy in Non-STEM (Fig. 1). Employing a computer-generated randomization process, teachers were allocated to one of these profiles. Each prompt included a profile picture accompanied by the inquiry: “A top-performing [boy or girl] student in your classroom would be impactful for others with respect to:” Participants were then prompted to rate, using a 0-100 scale (with zero reflecting no influence and 100 reflecting highest influence), the perceived impact of top-performing students across various domains including STEM performance, Non-STEM performance, classroom conduct, track selection in high school,^*^ college major choice, and occupational pursuits (additional details are provided in *Materials and Methods*). Profile pictures were carefully selected to subtly convey student excellence in different subjects while avoiding explicit influence on participants (43–45). The content displayed on the whiteboard in each picture was consistent for boys and girls within the same subject, ensuring experimental neutrality.^†^ Following exposure to a randomly assigned profile, teachers were promptly queried about their perceptions of the impact of top-performing boys or girls on their peers (*SI Appendix*, Figs. S6–S9).

## Results

### Respondent Characteristics.

Our analytical sample includes 670 responses.^‡^*SI Appendix*, Table S1 reports respondent demographics, specializations, personal educational experiences, explicit biases, and survey characteristics (i.e., survey timing, and duration).

Given the randomized assignment of student profiles, we anticipate no systematic discrepancies in their assignment to participants. Table 1 confirms the absence of such differences in a balancing test across the treatment conditions of Shown Boy (N: 333) and Shown Girl (N: 337).

**Table 1.** Balancing test across shown girl and shown boy conditions

	(1)	(2)	(3)	(4)	(5)	(6)
	Shown girl		Shown boy		Difference	
	Mean	SD	Mean	SD	Mean	SE
Demographics and history						
Female (1 = Yes)	0.82	0.38	0.81	0.39	0.01	0.03
Age (years)	41.30	10.35	41.23	10.72	0.06	0.88
Have a daughter (1 = Yes)	0.48	0.50	0.43	0.50	0.05	0.05
Have a son (1 = Yes)	0.48	0.50	0.43	0.50	0.05	0.05
Urban residence (1 = Yes)	0.56	0.50	0.53	0.50	0.03	0.04
Teacher’s specialization (1 = Yes):						
Preschool education	0.09	0.29	0.09	0.29	0.00	0.02
Primary school education	0.35	0.48	0.31	0.46	0.04	0.04
Secondary education:						
STEM subjects	0.17	0.38	0.19	0.39	-0.01	0.03
Social and humanitarian subjects	0.09	0.28	0.11	0.32	-0.03	0.03
Greek language	0.18	0.39	0.18	0.38	0.00	0.03
Foreign languages	0.12	0.32	0.12	0.33	-0.00	0.03
School years history (1 = Yes):						
Remember top performer’s gender	0.86	0.35	0.87	0.34	-0.01	0.03
Remember second best’s gender	0.61	0.49	0.57	0.50	0.04	0.04
Top performer was female	0.80	0.40	0.78	0.41	0.02	0.04
Second best was female	0.80	0.40	0.75	0.43	0.05	0.04
Was top or second best performer	0.24	0.43	0.22	0.42	0.02	0.03
Explicit biases						
Do you associate the following occupation with a specific gender? (1 = Yes)						
Engineer	0.24	0.43	0.22	0.42	0.02	0.04
Lawyer	0.04	0.20	0.04	0.20	0.00	0.02
Greek language teacher	0.14	0.35	0.10	0.30	0.04	0.03
Math teacher	0.12	0.33	0.08	0.27	0.04	0.03
Reported leniency toward female students (1 = Yes)	0.17	0.37	0.15	0.35	0.02	0.03
Survey characteristics						
Fall survey (1 = Yes)	0.27	0.44	0.20	0.40	0.07	0.03**
Duration (minutes)	7.72	2.94	7.37	2.97	0.35	0.23

The table reports summary statistics for teachers across the treatment conditions Shown Boy (N: 333) and Shown Girl (N: 337). Shown Girl refers to the treatment condition in which a participant was exposed to a profile of a top-performing girl. Shown Boy refers to a treatment condition in which a participant was exposed to a profile of a top-performing boy. STEM subjects include mathematics, physics, chemistry, biology, and computer science. Social and humanitarian subjects include theology, art, sociology, and economics. Greek language courses include Greek literature, language, and philosophy. ** indicates statistical significance from a two-sample mean comparison t test at the 5% level.

Columns 1 and 2 show the mean and SD for teachers exposed to a top-performing girl. Columns 3 and 4 report the mean and SD for teachers exposed to a top-performing boy. Column 5 presents the difference in means between columns 1 and 3. Column 6 shows the SEs. We find no statistically significant differences in participant or survey characteristics across the treatment conditions of Shown Boy and Shown Girl. We further conduct balancing tests across all four treatment scenarios (Shown Girl in STEM, Shown Boy in STEM, Shown Girl in Non-STEM, and Shown Boy in Non-STEM), with the results detailed in *SI Appendix*, Tables S2 and S3. The balancing tests show absence of statistically significant differences across all treatment conditions, corroborating the successful randomization process.

### Results by Top-Performer Gender.

**Fig. 2.**
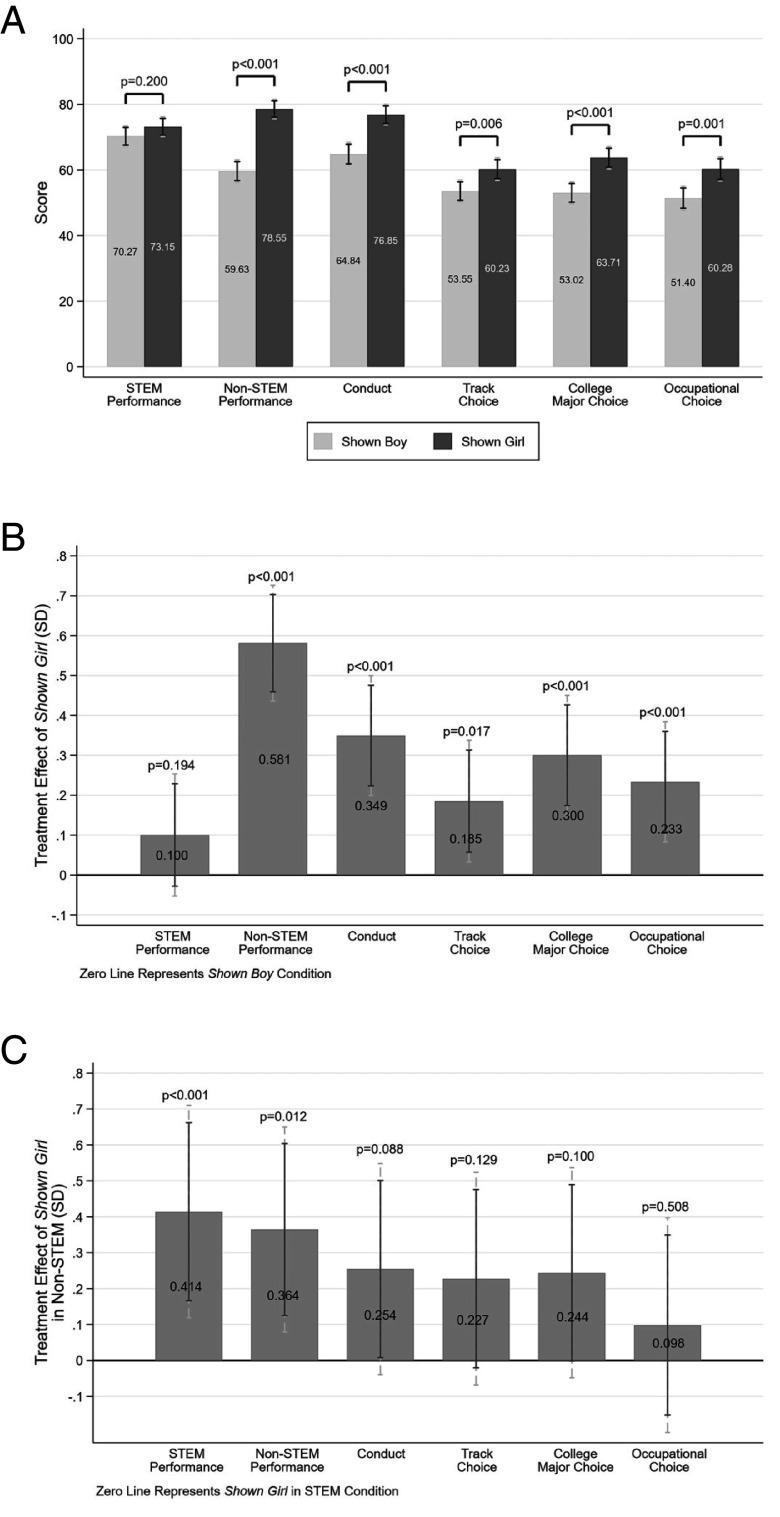
Teacher perceptions of role model influences of top performers. (*A*) presents the mean differences in teacher perceptions of role model influences of top-performing boys and girls across all outcomes. The y-axis values are raw scores with a range of [0 to 100]. *P*-values denote the significance levels from two sample mean comparison tests. (*B*) presents the estimated difference between the treatment conditions of Shown Girl and Shown Boy in SDs, controlling for participant and survey attributes (Eq. **1**). (*C*) presents the estimated coefficients of the interaction term between Shown Girl and Shown Non-STEM treatment conditions in SDs, controlling for participant and survey attributes (Eq. **2**). *P*-values correspond to tests of statistical significance of the estimated differences. Dashed and solid error bars represent 90% and 95% CIs, respectively.

Fig. 2*A* shows that teachers perceive both top-performing girls and boys as having a role model influence on their peers, with outcomes significantly different from zero. We find that teachers attribute greater role model influence to top-performing girls compared to top-performing boys in almost every outcome, including Non-STEM performance (P<0.001), conduct (79% vs. 60%; P<0.001), track choice (60% vs. 54%; P=0.006), college major choice (64% vs. 53%; P<0.001), and occupational pursuits (60% vs. 51%; P=0.001). There is no discernible difference in the perceived impact on other students’ STEM performance between top-performing girls and boys (73% vs. 70%; P=0.200).

We investigate the robustness of our results by controlling for a rich set of respondent and survey characteristics (Eq. **1** in *Material and Methods*). Fig. 2*B* reports our results in SD units. We find that even after adjusting for respondent characteristics, teachers associate top-performing girls with a significantly higher role model influence on other students’ Non-STEM performance ($\hat{\beta }=0.581$, SE=0.074, P<0.001), conduct ($\hat{\beta }=0.349$, SE=0.077, P<0.001), track choice ($\hat{\beta }=0.185$, SE=0.078, P=0.017), college major choice ($\hat{\beta }$= 0.300, SE= 0.076, P< 0.001), and occupational choice ($\hat{\beta }$= 0.233, SE= 0.077, P< 0.001) compared to top-performing boys.

We conduct a subsequent survey investigation of perceptions regarding study effort, attention to detail, diligence, and productivity associated to mitigate concerns about the amount of the whiteboard content displayed in the stimuli potentially priming respondents in favor of boys. We find no evidence of bias in favor of boys attributable to perceptions regarding study effort, attention to detail, diligence, or productivity (*SI Appendix*, Tables S16 and S17).

Taken together, these findings suggest that the average effect size (i.e., the difference between the Shown Girl and Shown Boy conditions) across all outcomes is 0.289 SD (SE= 0.058, P< 0.001).^§^ This indicates that teachers expect top-performing girls to have a greater influence on their peers, with a margin of 0.289 SDs compared to top-performing boys.

### Results by Top-Performer Gender and Subject Area.

We further explore our baseline findings to investigate whether teachers’ gender bias differs across academic domains (STEM vs. Non-STEM subjects). We do so by following two approaches. First, we estimate models including the interaction term between Shown Girl and Shown Non-STEM conditions (Eq. **2** in *Materials and Methods*). This approach allows us to estimate the STEM-specific gender bias relative to the Non-STEM-specific gender bias and gauge the statistical significance of their difference. *SI Appendix*, Table S5 presents the results controlling for participant and survey attributes. The marginal effect of the treatment condition Shown Girl in Non-STEM compared to the treatment condition Shown Boy in Non-STEM is given by the sum of the $\hat{\beta_{1}}$ and $\hat{\beta_{3}}$ coefficients. Regression results show that teachers perceive top-performing girls in Non-STEM as more impactful role models than top-performing boys in Non-STEM with respect to the outcome of STEM performance ($\hat{\beta_{1}}+\hat{\beta_{3}}=0.316$, SE=0.109, P=0.004), Non-STEM performance ($\hat{\beta_{1}}+\hat{\beta_{3}}=0.771$, SE=0.103, P<0.001), conduct ($\hat{\beta_{1}}+\hat{\beta_{3}}=0.482$, SE=0.109, P<0.001), track choice ($\hat{\beta_{1}}+\hat{\beta_{3}}=0.303$, SE=0.111, P=0.007), college major choice ($\hat{\beta_{1}}+\hat{\beta_{3}}=0.427$, SE=0.109, P<0.001), and occupational choice ($\hat{\beta_{1}}+\hat{\beta_{3}}=0.284$, SE=0.109, P=0.010).

Our regression results also show that teachers perceive top-performing girls in STEM as more impactful role models than top-performing boys in STEM. This difference is captured in the estimated coefficient $\hat{\beta_{1}}$ in Eq. **2**. Teachers perceive top-performing girls in STEM as significantly impactful role models with respect to the outcomes of Non-STEM performance ($\hat{\beta_{1}}$= 0.407, SE= 0.103, P< 0.001), conduct ($\hat{\beta_{1}}$= 0.228, SE= 0.105, P= 0.030), college major choice ($\hat{\beta_{1}}$= 0.184, SE= 0.104, P= 0.077), and occupational choice ($\hat{\beta_{1}}$= 0.186, SE= 0.107, P= 0.081).

We hypothesize that a girl excelling in STEM subjects is perceived as more likely to stand out and inspire her peers because her success contradicts the stereotype that girls are less likely to excel in STEM. However, the inspiration this girl generates may be perceived as more likely to translate into behavior that aligns with the stereotype-such as girls studying harder in Non-STEM subjects. This perceived consistency of influence from a standout girl in STEM on Non-STEM performance, rather than STEM performance, may result in a stronger overall signal (i.e., greater estimated effect) with tighter CIs for Non-STEM outcomes compared to STEM outcomes.

We also present how the marginal effects vary across gender (Girl vs. Boy) and subject (STEM vs. Non-STEM). Fig. 2*C* shows significant differences with respect to the outcome of STEM performance ($\hat{\beta }$= 0.414, SE= 0.150, P< 0.001), Non-STEM performance ($\hat{\beta }$= 0.364, SE= 0.145, P= 0.012), and conduct ($\hat{\beta }$= 0.254, SE= 0.150, P= 0.088).

Our second approach is based on a split-sample (heterogeneity) analysis that mirrors the baseline investigation in comparing teachers’ perceptions of the role model influence exerted by top-performing girls and boys. This approach produces similar estimates (*SI Appendix*, Figs. S1*A* and S2*A*) with our results remaining robust after controlling for respondent and survey characteristics (*SI Appendix*, Figs. S1*B* and S2*B*) (see *SI Appendix*, Table S4 for detailed results).

Overall, our results suggest that teachers’ gender bias differs across academic domains with the effects are more pronounced in terms of magnitude and precision for top-performing girls in Non-STEM subjects.

### Role Model Qualities.

We inquire into the qualities teachers perceive top performers who are successful role models to have. We focus on aspects such as confidence, learning autonomy, and sense of being an example for others. Recognizing that these traits may be associated with their status as exemplary students, we hypothesize that such characteristics could heighten their influence on other students.

**Fig. 3.**
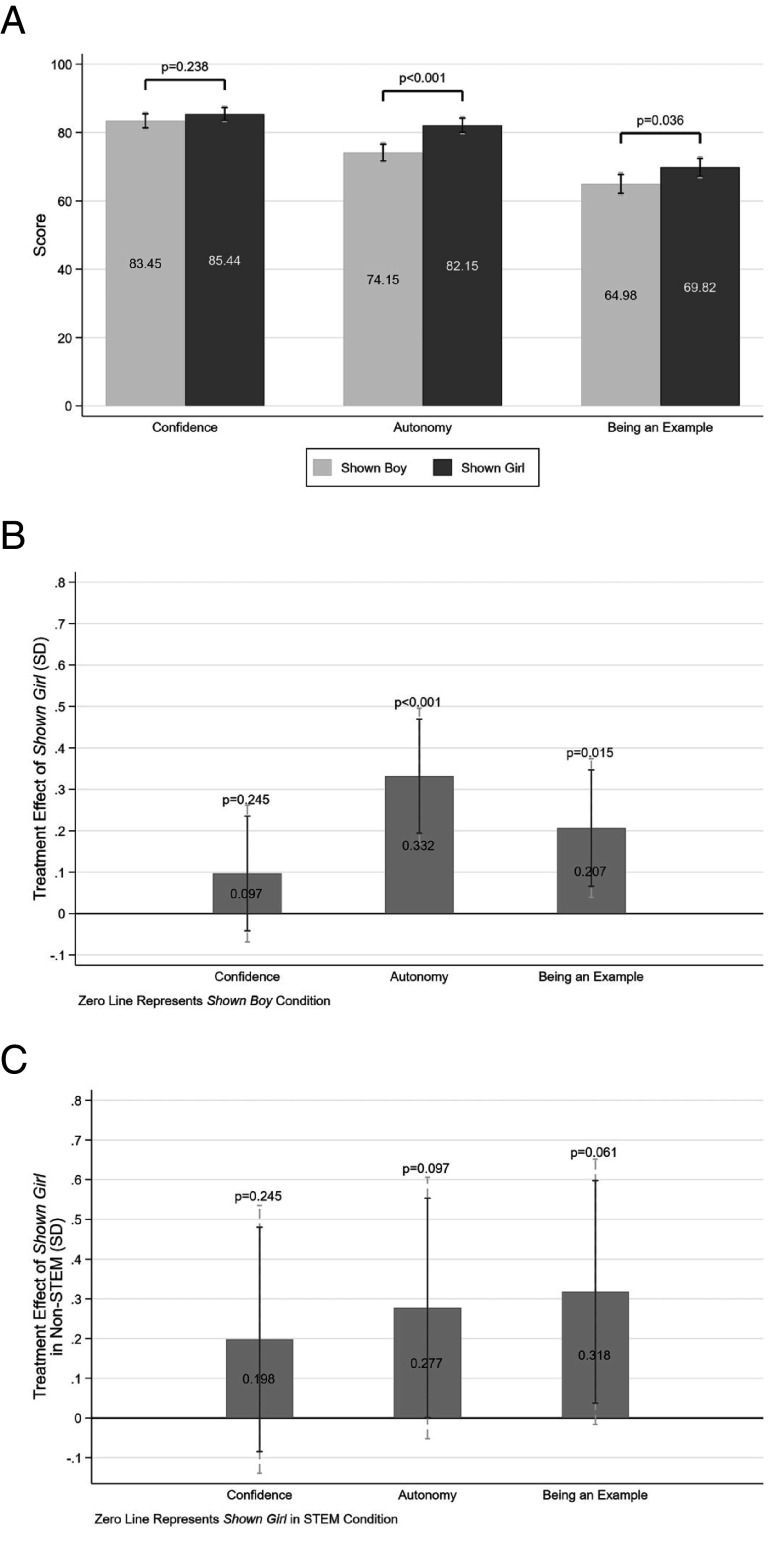
Perceived role model qualities. (*A*) presents the mean differences in the emotional conditions associated with role model influence teachers expect top-performing girls and boys to experience. The y-axis values are raw scores with a range of [0 to 100]. *P*-values denote the significance levels from two sample mean comparison tests. (*B*) presents the estimated difference between the treatment conditions of Shown Girl and Shown Boy in SDs, controlling for participant and survey attributes (Eq. **1**). (*C*) presents the estimated coefficients of the interaction term between Shown Girl and Shown Non-STEM treatment conditions in SDs, controlling for participant and survey attributes (Eq. **2**). *P*-values correspond to tests of statistical significance of the estimated differences. Dashed and solid error bars represent 90% and 95% CIs, respectively.

Overall, teachers perceive top-performing girls to have a greater sense of autonomy (P< 0.001) and sense of being an example (P= 0.036) relative to top-performing boys (Fig. 3*A*). We find no differences in how confident teachers expect top-performing girls and boys to feel. Our results remain robust when controlling for respondent and survey characteristics. Specifically, teachers expect the top-performing girls who are successful role models to experience a greater sense of autonomy ($\hat{\beta }$= 0.332, SE= 0.084, P< 0.001) and a greater sense of being an example ($\hat{\beta }$= 0.207, SE= 0.085, P= 0.015) relative to top-performing boys (Fig. 3*B*).^¶^

Among top performers in Non-STEM subjects, teachers perceive girls to possess greater learning autonomy ($\hat{\beta_{1}}$ + $\hat{\beta_{3}}$= 0.476, SE= 0.122, P< 0.001) and a greater sense of being an example for others ($\hat{\beta_{1}}$ + $\hat{\beta_{3}}$= 0.371, SE= 0.118, P= 0.002) (*SI Appendix*, Table S7). Among top performers in STEM subjects, teachers perceive girls to possess greater learning autonomy ($\hat{\beta_{1}}$= 0.199, SE= 0.114, P= 0.083). These differences remain when we use our split-sample approach (*SI Appendix*, Figs. S3 and S4; see *SI Appendix*, Table S6 for detailed results). Estimating the differences of these results, we find significant effects for the outcome of autonomy ($\hat{\beta }$= 0.277, SE= 0.168, P= 0.097) and sense of being an example ($\hat{\beta }$= 0.318, SE= 0.170, P= 0.061) (Fig. 3*C*).

These results suggest that teachers expect top-performing girls to feel more autonomous learners and more as an example for other students, which aligns with their perception of girls being more impactful to their peers.

### Results by Teacher Characteristics.

We conducted a heterogeneity analysis to investigate whether the baseline effects differ by teachers’ sex, age, parental status, and residential location. Results suggest that the effects are not statistically different between women (average $\hat{\beta }$= 0.322, SE= 0.065, P< 0.001) and men teachers (average $\hat{\beta }$= 0.256, SE= 0.130, P= 0.050) (*SI Appendix*, Table S8) or between teachers below (average $\hat{\beta }$= 0.250, SE= 0.096, P= 0.010) and above (average $\hat{\beta }$= 0.313, SE= 0.089, P< 0.001) the median age of 40 y (*SI Appendix*, Table S9). However, our estimates are more pronounced for teachers with children (average $\hat{\beta }$= 0.490, SE= 0.089, P< 0.001) than teachers without children (average $\hat{\beta }$= 0.204, SE= 0.106, P= 0.054) (*SI Appendix*, Table S10), and teachers residing in urban areas (average $\hat{\beta }$= 0.369, SE= 0.081, P< 0.001) than teachers residing in nonurban areas (average $\hat{\beta }$= 0.191, SE= 0.090, P= 0.035) (*SI Appendix*, Table S11). These results suggest that family life and social context may contribute to teachers’ gender stereotypes and consequently their perceptions regarding role model influences. These nuances highlight how teachers and students interact in complex ways, emphasizing the need to consider different demographic and social factors to fully understand these interactions.

### Statistical Bias.

Our survey results indicate that teachers perceive top-performing girls as more influential peer role models. One may worry that respondent beliefs reflect statistical realities in the study context, particularly if, on average, top-performing girls exhibit better academic and behavioral performance than their counterparts. To address this, we analyze hand-collected administrative transcript and attendance data sourced from the Greek Ministry of Education, covering a representative sample of 120 high schools.^#^ First, we use information on GPA and unexcused absences. Our investigation reveals limited evidence of differences in overall GPA between top-performing girls and boys (i.e., the highest performing student in each classroom). However, top-performing boys score slightly higher in STEM subjects, while top-performing girls score slightly higher in Non-STEM subjects (*SI Appendix*, Table S12, Panel *A*). This pattern also holds when examining the top 5% of students in each classroom (*SI Appendix*, Table S12, Panel *B*) instead of only focusing on the top performer in each classroom. We use unexcused absences as a behavioral proxy for conduct,^‖^ finding no statistically significant differences between top-performing girls and boys or between girls and boys in the top 5% of students in each classroom.

Next, we leverage information on double-blind exams conducted at the end of year 12 (17). We pursue this approach because actual performance differences, as reflected in GPA, could be influenced by teachers’ biases. At the end of high school, students take national exams that determine university admission. Exam papers with masked school and student information are graded by two external graders.^**^ Thus, national exam scores are as close as possible to “double-blind” since student’s name or gender is not directly revealed to graders and the students do not know the graders. These data enable us to examine differences in academic performance between top-performing boys and girls, minimizing the influence of potential teacher biases. We find that top-performing boys are either on par or outperform top-performing girls in every subject in double-blind exams with the exception of modern Greek (*SI Appendix*, Table S13). Overall performance differences, as reflected in GPA, favor girls, with their advantage in Non-STEM subjects surpassing boys’ edge in STEM (*SI Appendix*, Table S12). However, results from double-blind exams indicate that top-performing boys outperform top-performing girls in STEM subjects, with boys either matching or exceeding girls’ performance in all STEM-related subjects (*SI Appendix*, Table S13). Among, Non-STEM-related subjects top-performing girls outperform boys only in Modern Greek. These patterns hold for both top-performers and the top 5% of students in each class. Overall, these findings indicate that any statistical bias in teacher stereotypes may be limited.

### Empirical Evidence.

Our administrative data allow us to directly test whether top-performing girls have a greater impact on their classroom peers’ academic performance than top-performing boys. A unique feature of the Greek school system is that students are quasi-randomly (alphabetically) assigned to classrooms in the beginning of high school (grade 10), providing exogenous variation in the gender of the top performer in the classroom. Top performers in each classroom are officially recognized and are assigned the task of taking attendance during school day. Hence, one may anticipate that students randomly placed in classrooms with a top-performing girl will demonstrate higher performance compared to students in classrooms with a top-performing girl boy. We examine whether students in quasi-randomly formed classrooms in which the top performer is a girl have higher academic gains (i.e., difference between starting and final performance in grade 10) relative to classrooms in which the top performer is a boy (*SI Appendix*, Table S14). We find that students in classrooms with top-performing girls outperform their counterparts in classrooms with top-performing boys in terms of average end-of-year performance (P= 0.020) and academic gains (i.e., difference between end-of-year and start-of-year performance; P= 0.006). These empirical results provide supporting evidence to our survey-based experiment and are in line with reported teachers’ perceptions indicating that top-performing girls, in comparison with boys, exert greater positive influences on their classmates.

## Discussion

This study exploited a survey-based experiment to understand teacher beliefs on whether top-performing boys or girls can serve as role models for other students. Teacher attitudes toward potential peer role models are critical for the success of policies and interventions that rely on positive behavioral spillovers between students.

Our results show that top-performing girls are more likely to be perceived by teachers as impactful role models relative to top-performing boys. Our findings underscore the interplay between teachers’ gender stereotypes and perceived role model influences and qualities.

Valuable implications arise from our study. If teachers consistently perceive some students, even top-performing ones, as less likely to serve as role models, teachers may not encourage these students to embrace a role model function or may not encourage other students to view top performers as examples. This stance might limit the set of available role models in the classroom, potentially limiting the positive externalities between students (38). Promoting an open mind among teachers regarding who can be a role model might break vicious behavioral cycles and strengthen positive peer effects, leading to improved student outcomes for all (51, 52).

The gains from positive student interactions might be particularly valuable in resource-poor learning environments, that are often plagued by a general lack of high expectations and high-goal setting for students (53, 54). Understanding teachers’ beliefs in these contexts can contribute to the design of better initiatives (55). The gains of role models may also be rich among adolescents, since exposure to role models within existing social confines, such as peers, is typically much longer and has long-lasting effects (25).

Future research could focus on role model interventions in disadvantaged learning environments to better understand their potential in addressing poor aspirations, ultimately contributing to the design of more effective educational initiatives. Future research can also investigate the role of teachers in facilitating positive interactions and role model influences among peers.

Our approach in extracting teacher beliefs is general and can be applied in other contexts. Any research design aiming at eliciting stereotypes and biases may benefit from our teacher-focused survey experiment. The benefit of population-based survey experiments is that it allows for condition randomization across participant characteristics.

## Materials and Methods

### Randomized Survey Experiment Design.

We conducted a randomized controlled trial using online surveys involving teachers. Targeted population included teachers at all levels of education in Greece,^††^ whereas the average completion time of the questionnaire was 7 to 8 min. Participants were invited to give consent and take part in an incentivized survey about the influence of top-performing students on their fellow classmates (see, for example, *SI Appendix*, Fig. S10).

Initially, 705 responses were received. In consideration of survey quality, respondents who completed the survey in less than 2 min (N: 8) or more than 18 min (N: 27) were excluded from the analysis.^‡‡^ Consequently, our final analytical sample comprises 670 observations.

Each teacher who attempted the survey was randomly exposed to a profile of a top-performing student, accompanied by a photograph. Each profile clearly communicated the fact that 1) the student is a top performer, 2) the student’s gender, and 3) the subject area in which they excelled (STEM or Non-STEM subjects). Each participant was randomly exposed to one of the following treatment scenarios: top-performing girl in STEM (Profile A), top-performing girl in Non-STEM (Profile B), top-performing boy in STEM (Profile C), and top-performing boy in Non-STEM (Profile D) (Fig. 1).

Immediately after randomly exposing participants to the aforementioned profiles, we asked them to assess on a scale from 0 to 100 the degree to which a girl or boy student who excels in their class would be impactful for others with respect to: 1) STEM performance, 2) Non-STEM performance, 3) classroom conduct, 4) track selection in high school, 5) college major choice and 6) occupational choice. These six variables represent the main outcomes in our study (see *SI Appendix*, Figs. S6–S9 for examples of the randomized block questions). Subsequently, we inquired about the participants’ perceptions regarding the extent to which top-performer status for boys or girls in each profile is associated with specific behavioral explanations. We investigated behavioral explanations related to confidence, learning autonomy, sense of being an example for others. This allows us to understand the potential behavioral channels through which teachers perceive role model influences to operate.

Participants were also asked to recollect information and respond to questions about their actual top performer and second best student in class when they were students. Following this, participants were queried about their perceptions regarding gender associations with certain occupations, such as engineer, lawyer, language teacher, and math teacher. We also asked them whether they display greater leniency toward girls relative to boys in the classroom. These questions were formulated to assess any explicit biases among the participants. Last, we collected a comprehensive set of demographic characteristics from the respondents, including gender, age, parental status, residential location, and teaching specialization^§§^ (see *SI Appendix*, Fig. S11 for a comprehensive list of all survey questions presented in the sequential order they were asked).

### Empirical Strategy.

We estimate the effects of the survey experiment—i.e., the average effect of presenting a prompt describing a different treatment condition. The main specification is estimated as follows:

[1] $$Y_{\mathrm{ij}}=\beta_{0}+\beta_{1}Shown\;Girl_{i}+\gamma X_{\mathrm{ij}}+\theta +\tau +\epsilon_{\mathrm{ij}},$$

where $Y_{\mathrm{ij}}$ is the outcome variable measured after participant i has been exposed to a treatment condition j. We measure six different outcomes that capture the perceived influence of top performers on others with respect to: STEM performance, Non-STEM performance, conduct, track selection in high school, college major choice, and occupational choice. Variable Shown$Girl_{i}$ is a binary indicator that equals one if participant i has been randomly assigned to a profile of a top-performing girl j and zero otherwise. We account for the field of excellence of the top performer in the prompt by controlling for a binary indicator equal to one if participant *i* has been randomly exposed to a profile of a top-performing student *j* in STEM and zero otherwise. Vector $X_{\mathrm{ij}}$ captures respondents’ and survey characteristics. We also control for prefecture fixed effects (θ) and month fixed effects (τ) to account for spatial and time heterogeneity since participants were located at different prefectures in Greece and surveyed at different times.

Parameter $\beta_{1}$ reflects the coefficient of interest and captures the impact of exposure to a top-performing girl relative to a top-performing boy on teachers’ responses. Specification (Eq. **1**) is estimated using ordinary least squares and heteroskedasticity-robust SEs.

We also investigate the impact of exposure to a girl relative to a boy separately for top-performer profiles in STEM and Non-STEM. To do this, we estimate the following specification:

[2] $$\begin{array}{cc} Y_{\mathrm{ij}} & =\beta_{0}+\beta_{1}\;\text{Shown}\;{\text{Girl}}_{i}+\beta_{2}\;\text{Shown}\;{\text{Non-STEM}}_{i}\\ & \;+\beta_{3}\;\text{Shown}\;{\text{Girl}}_{i}\times \;\text{Shown}\;{\text{Non-STEM}}_{i}\\ & \;+\gamma X_{\mathrm{ij}}+\theta +\tau +\epsilon_{\mathrm{ij}}, \end{array}$$

where $Y_{\mathrm{ij}}$ denotes one of the aforementioned outcome variables measured after participant i has been exposed to a treatment condition j. Similarly to the specification Eq. **1**, Shown$Girl_{i}$ is a binary indicator that equals one if participant i has been randomly assigned to a profile of a top-performing girl j and zero otherwise. Shown$Non\text{-}STEM_{i}$ is a binary indicator that equals one if participant i has been randomly assigned to a profile of a top-performing student j in a Non-STEM subject and zero otherwise. We also include the interaction between Shown$Girl_{i}$ and Shown$Non\text{-}STEM_{i}$. All controls and fixed effects are the same as in specification Eq. **1**.^¶¶^

The estimated coefficient $\beta_{1}$ represents the treatment effect of teacher exposure to top-performing boys and girls in STEM. The linear combination of $\beta_{1}$ and $\beta_{3}$ coefficients estimates the treatment effect of teacher exposure to top-performing boys and girls in Non-STEM.

Alternatively, we follow a split-sample approach and run subsample regressions estimating specification (Eq. **1**) for STEM profiles (i.e., profile A vs. profile C) and Non-STEM profiles (i.e., profile B vs. profile D) separately. For instance, in *SI Appendix*, Fig. S1*B* the treatment variable is ShownGirlin$STEM_{i}$ and is a binary indicator equal to one if participant i has been randomly exposed to a profile of a top-performing girl j in STEM and zero otherwise. Similarly, in *SI Appendix*, Fig. S2*B* the treatment variable is ShownGirlinNon-$STEM_{i}$ and is a dummy equal to one if participant i has been randomly exposed to a profile of a top-performing girl j in Non-STEM and zero otherwise. This exercise is equivalent to estimating a model including interaction terms as in specification Eq. **2**.

.

## Supplementary Material

Appendix 01 (PDF)

## Data Availability

Anonymized participant-level data have been deposited in openICPSR (56).

## Footnotes

^*^Students in each track (i.e., Classics, Science, Information Technology) take a set of mandatory track-specific courses in addition to general education courses.

^†^*SI Appendix*, section S1 discusses results of an auxiliary survey conducted to investigate differential participants’ perceptions of role model influences because the whiteboard content may be perceived as indicator of hard work, effort, diligence, or attention to detail.

^‡^For details on the final sample construction, see *Materials and Methods*.

^§^We computed the average treatment effect size following Kling et al. (46). Let $\beta^{\mu }$ denote the estimated coefficient for the outcome variable μ, and $\sigma^{\mu }$ denote the SD of the same coefficient and outcome μ. The average treatment effect would be equal to $\frac{1}{M}\sum_{\mu =1}^{M}\frac{\beta^{\mu }}{\sigma^{\mu }}$, where M is the total number of outcome variables.

^¶^A mediation investigation (*SI Appendix*, Table S15) shows that teachers’ perceptions of top-performing girls’ sense of autonomy and their sense of being an example significantly mediate the influence of these girls as role models, compared to top-performing boys, across all outcomes. However, teachers’ perceptions of top-performing girls’ confidence do not significantly mediate their role model influence relative to boys in any outcome.

^#^This sample corresponds to roughly 10% of public schools in Greece. This is a representative sample of the population of high schools (30).

^‖^Attendance measures have been included in indicators of noncognitive skills in education (47, 48), whereas research has shown positive association between noncognitive skills and school conduct (49, 50). In our context, a student receives an unexcused absence when absent without a doctor’s or guardian’s note or when expelled from the classroom due to poor behavior. Also, we recognize that unexcused absences is an imperfect measure of the noncognitive skills necessary for positive role model influences. However, as technology becomes increasingly integrated into education and student communication shifts to digital platforms, we can expect more measures of attention, engagement, extracurricular participation, collaboration, and social skills to emerge.

^**^Graders are teachers in other schools and are usually in different parts of the country than the students.

^††^While pre-K, Kindergarten, and primary school teachers have pedagogical but no subject-matter expertise, secondary education (i.e., middle and high school) teachers are specialized in specific fields based on their college education.

^‡‡^Our results do not change if we include these observations in the analytical sample.

^§§^While preschool and primary school teachers cover all subjects, secondary school (high school) teachers are specialized in specific fields.

^¶¶^In both Eqs. **1** and **2**, we include binary indicators for missing values in the covariates.
