# Supplementary material for: Top-performing girls are more impactful peer role models than boys, teachers say
Source: Proc Natl Acad Sci U S A. 2025 Feb 6;122(6):e2421436122. doi: 10.1073/pnas.2421436122 (PMC11831187; doi:10.1073/pnas.2421436122)
Supplement: Supplementary file 1 — Appendix 01 (PDF) [file pnas.2421436122.sapp.pdf]

Supplementary Appendix

Top-performing Girls are more Impactful Peer Role Models than Boys, Teachers Say

Table S1: Respondent Characteristics

	(1) Mean	(2) SD	(3) Min	(4) Max
Demographics and History				
Female (1=Yes)	0.82	0.38	0	1
Age (Years)	41.27	10.53	22	65
Have a Daughter (1=Yes)	0.45	0.50	0	1
Have a Son (1=Yes)	0.46	0.50	0	1
Urban Residence (1=Yes)	0.54	0.50	0	1
Teacher's Specialization (1=Yes):				
Pre-school Education	0.09	0.29	0	1
Primary School Education	0.33	0.47	0	1
Secondary Education:				
STEM Subjects	0.18	0.38	0	1
Social & Humanitarian Subjects	0.10	0.30	0	1
Greek Language	0.18	0.38	0	1
Foreign Languages	0.12	0.33	0	1
School Years History (1=Yes):				
Remember Top Performer's Gender	0.86	0.35	0	1
Remember Second Best's Gender	0.59	0.49	0	1
Top Performer was Female	0.79	0.41	0	1
Second Best was Female	0.78	0.42	0	1
Was Top or Second Best Performer	0.23	0.42	0	1
Explicit Biases				
Do you Associate the Following Occupation with a Specific Gender? (1=Yes)				
Engineer	0.23	0.42	0	1
Lawyer	0.04	0.20	0	1
Greek Language Teacher	0.12	0.33	0	1
Math Teacher	0.10	0.30	0	1
Reported Leniency Toward Female Students (1=Yes)	0.16	0.36	0	1
Survey Characteristics				
Fall Survey (1=Yes)	0.23	0.42	0	1
Duration (Minutes)	7.54	2.96	2	18

Notes: The table reports descriptive statistics for teachers' demographic characteristics and school years history, explicit biases and survey's characteristics. Sample consists of 670 teachers. Teachers specializing in STEM subjects include those who teach mathematics, physics, chemistry, biology, and computer science; specializing in social & humanitarian encompasses those who instruct in theology, art, sociology, and economics; specializing in Greek language includes those who teach Greek literature, language, and philosophy.

Table S2: Balancing Test Across Shown Girl in STEM and Shown Boy in STEM Conditions

	(1)	(2)	(3)	(4)	(5)	(6)
	Shown Girl in STEM		Shown Boy in STEM		Difference (1) - (3)	
	Mean	SD	Mean	SD	Mean	SE
Demographics and History						
Female (1=Yes)	0.84	0.37	0.82	0.38	0.02	0.04
Age (Years)	40.34	10.14	41.35	10.55	-1.01	1.20
Have a Daughter (1=Yes)	0.50	0.50	0.41	0.49	0.09	0.08
Have a Son (1=Yes)	0.51	0.50	0.45	0.50	0.05	0.07
Urban Residence (1=Yes)	0.58	0.50	0.53	0.50	0.05	0.06
Teacher's Specialization (1=Yes):						
Pre-school Education	0.10	0.30	0.10	0.30	0.00	0.04
Primary School Education	0.36	0.48	0.28	0.45	0.08	0.06
Secondary Education:						
STEM Subjects	0.16	0.37	0.20	0.40	-0.04	0.05
Social & Humanitarian Subjects	0.09	0.29	0.11	0.31	-0.01	0.04
Greek Language	0.19	0.39	0.20	0.40	-0.01	0.05
Foreign Languages	0.10	0.30	0.11	0.32	-0.01	0.04
School Years History (1=Yes):						
Remember Top Performer's Gender	0.86	0.35	0.85	0.36	0.01	0.04
Remember Second Best's Gender	0.58	0.49	0.57	0.50	0.01	0.06
Top Performer was Female	0.79	0.41	0.75	0.43	0.04	0.05
Second Best was Female	0.79	0.41	0.75	0.44	0.04	0.06
Was Top or Second Best Performer	0.24	0.43	0.23	0.42	0.01	0.05
Explicit Biases						
Do you Associate the Following Occupation with a Specific Gender? (1=Yes)						
Engineer	0.26	0.44	0.23	0.42	0.03	0.06
Lawyer	0.07	0.25	0.06	0.24	0.01	0.03
Greek Language Teacher	0.17	0.38	0.10	0.30	0.08	0.05*
Math Teacher	0.13	0.34	0.09	0.28	0.04	0.04
Reported Leniency Toward Female Students (1=Yes)	0.18	0.38	0.17	0.38	0.01	0.04
Survey Characteristics						
Fall Survey (1=Yes)	0.25	0.43	0.21	0.41	0.04	0.05
Duration (Minutes)	7.58	2.87	7.23	2.92	0.36	0.31

Notes: The table reports descriptive statistics for teachers across the treatment groups *Shown Boy in STEM* (N: 173) and *Shown Girl in STEM* (N: 174). *Shown Girl in STEM* refers to a treatment scenario where the participant was exposed to a top-performing girl in STEM. *Shown Boy in STEM* refers to a treatment scenario where the participant was exposed to a top-performing boy in STEM. Teachers specializing in STEM subjects include those who teach mathematics, physics, chemistry, biology, and computer science; specializing in social & humanitarian encompasses those who instruct in theology, art, sociology, and economics; specializing in Greek language includes those who teach Greek literature, language, and philosophy. Significance stars denote the results from two sample mean comparison t-tests; *, ** and *** indicate statistical significance at the 10%, 5% and 1% level, respectively.

Table S3: Balancing Test Across Shown Girl in Non-STEM and Shown Boy in Non-STEM Conditions

	(1)	(2)	(3)	(4)	(5)	(6)
	Shown Girl in Non-STEM		Shown Boy in Non-STEM		Difference (1) - (3)	
	Mean	SD	Mean	SD	Mean	SE
Demographics and History						
Female (1=Yes)	0.81	0.39	0.81	0.40	0.00	0.04
Age (Years)	42.35	10.50	41.12	10.94	1.23	1.28
Have a Daughter (1=Yes)	0.46	0.50	0.44	0.50	0.01	0.07
Have a Son (1=Yes)	0.45	0.50	0.40	0.49	0.05	0.07
Urban Residence (1=Yes)	0.54	0.50	0.53	0.50	0.01	0.06
Teacher's Specialization (1=Yes):						
Pre-school Education	0.09	0.28	0.08	0.27	0.01	0.03
Primary School Education	0.34	0.47	0.34	0.47	-0.00	0.06
Secondary Education:						
STEM Subjects	0.18	0.39	0.17	0.38	0.01	0.05
Social & Humanitarian Subjects	0.08	0.27	0.12	0.33	-0.04	0.04
Greek Language	0.18	0.38	0.16	0.37	0.02	0.05
Foreign Languages	0.14	0.35	0.14	0.34	0.00	0.04
School Years History (1=Yes):						
Remember Top Performer's Gender	0.85	0.35	0.88	0.33	-0.03	0.04
Remember Second Best's Gender	0.64	0.48	0.57	0.50	0.07	0.06
Top Performer was Female	0.82	0.39	0.82	0.39	0.00	0.05
Second Best was Female	0.81	0.39	0.75	0.43	0.06	0.06
Was Top or Second Best Performer	0.24	0.43	0.22	0.41	0.02	0.05
Explicit Biases						
Do you Associate the Following Occupation with a Specific Gender? (1=Yes)						
Engineer	0.22	0.41	0.21	0.41	0.01	0.06
Lawyer	0.02	0.13	0.03	0.17	-0.01	0.02
Greek Language Teacher	0.11	0.31	0.10	0.31	0.00	0.04
Math Teacher	0.11	0.31	0.08	0.27	0.03	0.04
Reported Leniency Toward Female Students (1=Yes)	0.15	0.36	0.12	0.32	0.03	0.04
Survey Characteristics						
Fall Survey (1=Yes)	0.29	0.45	0.18	0.39	0.11	0.05**
Duration (Minutes)	7.86	3.01	7.53	3.03	0.33	0.34

Notes: The table reports descriptive statistics for teachers across the treatment groups *Shown Boy in Non-STEM* (N: 160) and *Shown Girl in Non-STEM* (N: 163). *Shown Girl in Non-STEM* refers to a treatment scenario where the participant was exposed to a top-performing girl in Non-STEM. *Shown Boy in Non-STEM* refers to a treatment scenario where the participant was exposed to a top-performing boy in Non-STEM. Teachers specializing in STEM subjects include those who teach mathematics, physics, chemistry, biology, and computer science; specializing in social & humanitarian encompasses those who instruct in theology, art, sociology, and economics; specializing in Greek language includes those who teach Greek literature, language, and philosophy. Significance stars denote the results from two sample mean comparison t-tests; *, ** and *** indicate statistical significance at the 10%, 5% and 1% level, respectively.

Table S4: Treatment Effect of Teacher Exposure to Top Performers

	(1) STEM Performance	(2) Non-STEM Performance	(3) Conduct	(4) Track Choice	(5) College Major Choice	(6) Occupational Choice
Panel A: Girl Relative to Boy						
Shown Girl	0.100	0.581***	0.349***	0.185**	0.300***	0.233***
	(0.078)	(0.074)	(0.077)	(0.078)	(0.076)	(0.077)
Shown STEM	Yes	Yes	Yes	Yes	Yes	Yes
Controls	Yes	Yes	Yes	Yes	Yes	Yes
Prefecture Fixed Effects	Yes	Yes	Yes	Yes	Yes	Yes
Month Fixed Effects	Yes	Yes	Yes	Yes	Yes	Yes
Observations	670	670	670	670	670	670
R-squared	0.111	0.189	0.132	0.148	0.174	0.161
Panel B: Girl Relative to Boy in STEM						
Shown Girl in STEM	-0.102	0.393***	0.240**	0.067	0.165	0.183*
	(0.108)	(0.105)	(0.107)	(0.108)	(0.107)	(0.110)
Controls	Yes	Yes	Yes	Yes	Yes	Yes
Prefecture Fixed Effects	Yes	Yes	Yes	Yes	Yes	Yes
Month Fixed Effects	Yes	Yes	Yes	Yes	Yes	Yes
Observations	347	347	347	347	347	347
R-squared	0.177	0.211	0.160	0.222	0.253	0.226
Panel C: Girl Relative to Boy in Non-STEM						
Shown Girl in non-STEM	0.338***	0.792***	0.514***	0.311***	0.444***	0.317***
	(0.113)	(0.106)	(0.112)	(0.116)	(0.115)	(0.113)
Controls	Yes	Yes	Yes	Yes	Yes	Yes
Prefecture Fixed Effects	Yes	Yes	Yes	Yes	Yes	Yes
Month Fixed Effects	Yes	Yes	Yes	Yes	Yes	Yes
Observations	323	323	323	323	323	323
R-squared	0.198	0.278	0.213	0.189	0.206	0.224

Notes: Outcomes are standardized to have a mean equal to zero and a standard deviation equal to one. Panel A estimates treatment effects of teacher exposure to top-performing girls and boys irrespective of the subject these top performers excelled in with a *Shown STEM* binary indicator being included in all specifications. Panel B estimates treatment effects of teacher exposure to top-performing girls and boys in STEM. Panel C estimates treatment effects of teacher exposure to top-performing girls and boys in Non-STEM. In all specifications we control for demographics, teacher specializations, own history from school years, explicit biases, survey characteristics and state, month fixed effects. We control for indicators reflecting any missing values. Robust standard errors in parentheses; *, ** and *** indicate statistical significance at the 10%, 5% and 1% level, respectively.

Table S5: Treatment Effect of Teacher Exposure to Top Performers: Specifications Using Interaction Terms

	(1) STEM Performance	(2) Non-STEM Performance	(3) Conduct	(4) Track Choice	(5) College Major Choice	(6) Occupational Choice
Shown Girl [β_1]	-0.098	0.407***	0.228**	0.076	0.184*	0.186*
	(0.106)	(0.103)	(0.105)	(0.105)	(0.104)	(0.107)
Shown Non-STEM [β_2]	-0.269**	-0.304***	-0.191*	-0.157	-0.060	-0.059
	(0.109)	(0.112)	(0.115)	(0.106)	(0.108)	(0.108)
Shown Girl x Shown Non-STEM [β_3]	0.414***	0.364**	0.254*	0.227	0.244	0.098
	(0.150)	(0.145)	(0.150)	(0.151)	(0.149)	(0.152)
Observations	670	670	670	670	670	670
R-squared	0.121	0.197	0.136	0.151	0.178	0.162
P-value [β_1] + [β_3]	0.004	0.000	0.000	0.007	0.000	0.010
Controls	Yes	Yes	Yes	Yes	Yes	Yes
Prefecture Fixed Effects	Yes	Yes	Yes	Yes	Yes	Yes
Month Fixed Effects	Yes	Yes	Yes	Yes	Yes	Yes

Notes: Outcomes are standardized to have a mean equal to zero and a standard deviation equal to one. In all specifications we control for demographics, teacher specializations, own history from school years, explicit biases, survey characteristics and state, month fixed effects. We control for indicators reflecting any missing values. Robust standard errors in parentheses; *, ** and *** indicate statistical significance at the 10%, 5% and 1% level, respectively.

Table S6: Behavioral Channels Associated with Role Model Influences

	(1) Confidence	(2) Autonomy	(3) Being an Example
Panel A: Girl Relative to Boy			
Shown Girl	0.097	0.332***	0.207**
	(0.084)	(0.084)	(0.085)
Shown STEM	Yes	Yes	Yes
Controls	Yes	Yes	Yes
Prefecture Fixed Effects	Yes	Yes	Yes
Month Fixed Effects	Yes	Yes	Yes
Observations	578	576	568
R-squared	0.070	0.122	0.113
Panel B: Girl Relative to Boy in STEM			
Shown Girl in STEM	0.022	0.207*	0.032
	(0.115)	(0.117)	(0.127)
Controls	Yes	Yes	Yes
Prefecture Fixed Effects	Yes	Yes	Yes
Month Fixed Effects	Yes	Yes	Yes
Observations	298	297	291
R-squared	0.099	0.121	0.121
Panel C: Girl Relative to Boy in Non-STEM			
Shown Girl in non-STEM	0.214	0.507***	0.438***
	(0.135)	(0.130)	(0.124)
Controls	Yes	Yes	Yes
Prefecture Fixed Effects	Yes	Yes	Yes
Month Fixed Effects	Yes	Yes	Yes
Observations	280	279	277
R-squared	0.148	0.237	0.213

Notes: Outcomes are standardized to have a mean equal to zero and a standard deviation equal to one. Panel A estimates treatment effects of teacher exposure to top-performing girls and boys irrespective of the subject these top performers excelled in with a *Shown STEM* binary indicator being included in all specifications. Panel B estimates treatment effects of teacher exposure to top-performing girls and boys in STEM. Panel C estimates treatment effects of teacher exposure to top-performing girls and boys in Non-STEM. In all specifications we control for demographics, teacher specializations, own history from school years, explicit biases, survey characteristics and state, month fixed effects. We control for indicators reflecting any missing values. Robust standard errors in parentheses; *, ** and *** indicate statistical significance at the 10%, 5% and 1% level, respectively.

Table S7: Behavioral Channels Associated with Role Model Influences: Specifications Using Interaction Terms

	(1)<br>Confidence	(2)<br>Autonomy	(3)<br>Being an Example
Shown Girl [β_1]	0.002	0.199*	0.053
	(0.114)	(0.114)	(0.122)
Shown Non-STEM [β_2]	-0.164	-0.177	0.016
	(0.129)	(0.128)	(0.124)
Shown Girl x Shown Non-STEM [β_3]	0.198	0.277*	0.318*
	(0.172)	(0.168)	(0.170)
Observations	578	576	568
R-squared	0.072	0.126	0.119
P-value [β_1] + [β_3]	0.115	0.000	0.002
Controls	Yes	Yes	Yes
Prefecture Fixed Effects	Yes	Yes	Yes
Month Fixed Effects	Yes	Yes	Yes

Notes: Outcomes are standardized to have a mean equal to zero and a standard deviation equal to one. In all specifications we control for demographics, teacher specializations, own history from school years, explicit biases, survey characteristics and state, month fixed effects. We control for indicators reflecting any missing values. Robust standard errors in parentheses; *, ** and *** indicate statistical significance at the 10%, 5% and 1% level, respectively.

Table S8: Heterogeneity Analysis: Subsample Regressions by Teacher's Sex

	(1) STEM Performance	(2) Non-STEM Performance	(3) Conduct	(4) Track Choice	(5) College Major Choice	(6) Occupational Choice
Panel A: *Women Teachers*						
[1] Shown Girl	0.126	0.597***	0.413***	0.187**	0.320***	0.289***
	(0.089)	(0.083)	(0.087)	(0.087)	(0.085)	(0.085)
Observations	549	549	549	549	549	549
R-squared	0.121	0.202	0.160	0.150	0.187	0.173
Panel B: *Men Teachers*						
[2] Shown Girl	0.138	0.552***	0.238	0.184	0.315	0.163
	(0.203)	(0.188)	(0.195)	(0.211)	(0.207)	(0.203)
Observations	121	121	121	121	121	121
R-squared	0.360	0.410	0.326	0.428	0.379	0.390
P-value Diff [1]-[2]	0.947	0.789	0.319	0.988	0.978	0.486
Shown STEM	Yes	Yes	Yes	Yes	Yes	Yes
Controls	Yes	Yes	Yes	Yes	Yes	Yes
Prefecture Fixed Effects	Yes	Yes	Yes	Yes	Yes	Yes
Month Fixed Effects	Yes	Yes	Yes	Yes	Yes	Yes

Notes: Outcomes are standardized to have a mean equal to zero and a standard deviation equal to one. Panel A estimates treatment effects of teacher exposure to top-performing girls and boys for women teachers, whereas Panel B focuses on men teachers. In all specifications we control for demographics, teacher specializations, own history from school years, explicit biases, survey characteristics and state, month fixed effects. We control for indicators reflecting any missing values. Robust standard errors in parentheses; *, ** and *** indicate statistical significance at the 10%, 5% and 1% level, respectively.

Table S9: Heterogeneity Analysis: Subsample Regressions by Teacher's Age

	(1) STEM Performance	(2) Non-STEM Performance	(3) Conduct	(4) Track Choice	(5) College Major Choice	(6) Occupational Choice
Panel A: *Age* $<$ *40*						
[1] Shown Girl	-0.003	0.575***	0.439***	0.027	0.193	0.167
	(0.120)	(0.122)	(0.117)	(0.130)	(0.128)	(0.127)
Observations	273	273	273	273	273	273
R-squared	0.155	0.221	0.203	0.206	0.225	0.204
Panel B: *Age* $\geq$ *40*						
[2] Shown Girl	0.170	0.573***	0.342***	0.268**	0.342***	0.292**
	(0.129)	(0.121)	(0.128)	(0.123)	(0.121)	(0.119)
Observations	304	304	304	304	304	304
R-squared	0.136	0.222	0.170	0.208	0.222	0.245
P-value Diff [1]-[2]	0.290	0.990	0.546	0.147	0.360	0.437
Shown STEM	Yes	Yes	Yes	Yes	Yes	Yes
Controls	Yes	Yes	Yes	Yes	Yes	Yes
Prefecture Fixed Effects	Yes	Yes	Yes	Yes	Yes	Yes
Month Fixed Effects	Yes	Yes	Yes	Yes	Yes	Yes

Notes: Outcomes are standardized to have a mean equal to zero and a standard deviation equal to one. Panel A estimates treatment effects of teacher exposure to top-performing girls and boys for teachers aged less than 40, whereas Panel B focuses on teachers aged 40 and above. In all specifications we control for demographics, teacher specializations, own history from school years, explicit biases, survey characteristics and state, month fixed effects. We control for indicators reflecting any missing values. Robust standard errors in parentheses; *, ** and *** indicate statistical significance at the 10%, 5% and 1% level, respectively.

Table S10: Heterogeneity Analysis: Subsample Regressions by Teacher's Parental Status

	(1) STEM Performance	(2) Non-STEM Performance	(3) Conduct	(4) Track Choice	(5) College Major Choice	(6) Occupational Choice
Panel A: *With Children*						
[1] Shown Girl	0.323**	0.739***	0.364***	0.562***	0.600***	0.443***
	(0.125)	(0.120)	(0.123)	(0.125)	(0.124)	(0.129)
Observations	243	243	243	243	243	243
R-squared	0.207	0.294	0.197	0.234	0.247	0.229
Panel B: *Without Children*						
[2] Shown Girl	-0.103	0.605***	0.493***	-0.080	0.145	0.099
	(0.142)	(0.141)	(0.133)	(0.127)	(0.125)	(0.142)
Observations	197	197	197	197	197	197
R-squared	0.194	0.221	0.253	0.177	0.191	0.198
P-value Diff [1]-[2]	0.013	0.421	0.432	0.000	0.004	0.048
Shown STEM	Yes	Yes	Yes	Yes	Yes	Yes
Controls	Yes	Yes	Yes	Yes	Yes	Yes
Prefecture Fixed Effects	Yes	Yes	Yes	Yes	Yes	Yes
Month Fixed Effects	Yes	Yes	Yes	Yes	Yes	Yes

Notes: Outcomes are standardized to have a mean equal to zero and a standard deviation equal to one. Panel A estimates treatment effects of teacher exposure to top-performing girls and boys for teachers who have kids, whereas Panel B focuses on teachers who don't have kids. In all specifications we control for demographics, teacher specializations, own history from school years, explicit biases, survey characteristics and state, month fixed effects. We control for indicators reflecting any missing values. Robust standard errors in parentheses; *, ** and *** indicate statistical significance at the 10%, 5% and 1% level, respectively.

Table S11: Heterogeneity Analysis: Sub-sample Regressions by Teacher's Locale

	(1) STEM Performance	(2) Non-STEM Performance	(3) Conduct	(4) Track Choice	(5) College Major Choice	(6) Occupational Choice
Panel A: *Urban Locale*						
[1] Shown Girl	0.156	0.525***	0.478***	0.351***	0.434***	0.369***
	(0.125)	(0.116)	(0.118)	(0.121)	(0.117)	(0.115)
Observations	319	319	319	319	319	319
R-squared	0.175	0.223	0.233	0.238	0.249	0.241
Panel B: *Non-urban Locale*						
[2] Shown Girl	0.019	0.645***	0.272**	-0.028	0.101	0.116
	(0.121)	(0.114)	(0.118)	(0.121)	(0.123)	(0.126)
Observations	267	267	267	267	267	267
R-squared	0.150	0.283	0.169	0.216	0.234	0.223
P-value Diff [1]-[2]	0.398	0.426	0.183	0.016	0.034	0.110
Shown STEM	Yes	Yes	Yes	Yes	Yes	Yes
Controls	Yes	Yes	Yes	Yes	Yes	Yes
Prefecture Fixed Effects	Yes	Yes	Yes	Yes	Yes	Yes
Month Fixed Effects	Yes	Yes	Yes	Yes	Yes	Yes

Notes: Outcomes are standardized to have a mean equal to zero and a standard deviation equal to one. Panel A estimates treatment effects of teacher exposure to top-performing girls and boys for teachers who reside in urban areas, whereas Panel B focuses on teachers who reside in rural areas. In all specifications we control for demographics, teacher specializations, own history from school years, explicit biases, survey characteristics and state, month fixed effects. We control for indicators reflecting any missing values. Robust standard errors in parentheses; *, ** and *** indicate statistical significance at the 10%, 5% and 1% level, respectively.

Table S12: Performance and Behavior Differences of Top Performing Students

	(1)	(2)	(3)	(4)	(5)	(6)
	Girls		Boys		Difference (1) - (3)	
	Mean	SD	Mean	SD	Mean	SE
**Panel A: Top Performing Students**						
Overall Performance						
Grade 10	18.92	0.78	18.86	0.81	0.05	0.03**
Grade 11	18.83	0.95	18.79	0.99	0.04	0.03
Grade 12	18.90	0.78	18.89	0.95	0.00	0.03
STEM Performance						
Grade 10	18.76	1.08	18.97	0.97	-0.21	0.03***
Grade 11	18.91	1.12	19.12	0.92	-0.21	0.03***
Grade 12	18.90	1.08	19.15	1.06	-0.24	0.03***
Non-STEM Performance						
Grade 10	18.86	0.82	18.59	0.99	0.27	0.03***
Grade 11	18.75	0.95	18.38	1.21	0.37	0.04***
Grade 12	18.94	0.78	18.70	1.09	0.25	0.03***
Unexcused Absences						
Grade 10	14.68	10.84	13.93	10.45	0.75	0.45*
Grade 11	19.12	12.19	18.12	12.27	1.00	0.55*
Grade 12	18.83	13.50	19.26	13.70	-0.43	1.00
**Panel B: Top 5% of Students**						
Overall Performance						
Grade 10	18.73	0.83	18.66	0.86	0.07	0.02***
Grade 11	18.68	0.95	18.64	0.99	0.04	0.03
Grade 12	18.77	0.78	18.75	0.92	0.02	0.02
STEM Performance						
Grade 10	18.52	1.19	18.75	1.10	-0.23	0.03***
Grade 11	18.74	1.16	18.98	1.00	-0.24	0.03***
Grade 12	18.73	1.15	19.04	1.03	-0.31	0.03***
Non-STEM Performance						
Grade 10	18.69	0.87	18.39	1.03	0.30	0.02***
Grade 11	18.62	0.97	18.22	1.22	0.40	0.03***
Grade 12	18.85	0.81	18.53	1.09	0.32	0.03***
Unexcused Absences						
Grade 10	15.18	11.23	14.62	10.74	0.56	0.36
Grade 11	19.67	12.40	18.95	12.44	0.72	0.45
Grade 12	19.90	13.90	20.64	14.03	-0.74	0.81

Notes: The table presents summary statistics produced utilizing administrative data from Greece. Sample consists of 4,772 top-performing girls and 2,437 top-performing boys in their own class/grade from 123 schools, corresponding to 10% of the total number of public schools. Panel A identifies top performing students as those with the highest overall performance in each classroom. Panel B identifies the top 5% of students with the highest overall performance in each classroom. Student performance is measured on a scale between 0 and 20. Unexcused absences are a continuous variable and serve as a behavioral proxy, as students receive an unexcused absence when the teacher dismisses them from the class due to poor conduct. *, ** and *** indicate statistical significance at the 10%, 5% and 1% level, respectively.

Table S13: Performance Differences of Top Performing Students in Blind Exams

	(1)	(2)	(3)	(4)	(5)	(6)
	Girls		Boys		Difference (1) - (3)	
	Mean	SD	Mean	SD	Mean	SE
**Panel A: Top Performing Students**						
Ancient Greek	17.18	2.06	17.35	2.12	-0.17	0.18
Biology	18.27	1.57	18.33	1.73	-0.06	0.08
Chemistry	18.73	1.60	18.86	1.58	-0.14	0.08*
Computer Programming	18.09	2.12	18.67	1.92	-0.58	0.12***
Greek Literature	16.59	2.26	16.61	2.15	-0.02	0.18
History	18.02	2.24	18.27	1.88	-0.25	0.16
Latin	18.63	1.66	18.68	1.68	-0.06	0.14
Advanced Mathematics	16.45	2.97	17.18	2.87	-0.72	0.11***
Mathematics and Statistics	17.93	3.34	18.84	2.33	-0.92	0.09***
Advanced Physics	16.92	3.07	17.78	2.58	-0.86	0.10***
Modern Greek	16.01	1.78	15.67	1.95	0.34	0.06***
**Panel B: Top 5% of Students**						
Ancient Greek	16.98	2.07	17.22	1.95	-0.25	0.13*
Biology	18.09	1.65	18.10	1.79	-0.02	0.07
Chemistry	18.48	1.83	18.70	1.72	-0.22	0.07***
Computer Programming	17.95	2.14	18.46	2.02	-0.52	0.09***
Greek Literature	16.43	2.27	16.44	2.17	-0.01	0.14
History	17.87	2.27	18.27	1.80	-0.41	0.12***
Latin	18.50	1.67	18.55	1.67	-0.05	0.11
Advanced Mathematics	16.11	3.08	16.86	2.95	-0.75	0.09***
Mathematics and Statistics	17.54	3.64	18.75	2.38	-1.21	0.07***
Advanced Physics	16.64	3.12	17.47	2.75	-0.83	0.09***
Modern Greek	15.85	1.83	15.45	1.96	0.40	0.05***

Notes: The table presents summary statistics produced utilizing administrative data on blind exams in grade 12. Sample consists of 4,475 top-performing girls and 2,313 top-performing boys in their own class/grade from 123 schools, corresponding to 10% of the total number of public schools. Panel A identifies top performing students as those with the highest overall performance in each classroom. Panel B identifies the top 5% of students with the highest overall performance in each classroom. Student performance is measured on a scale between 0 and 20. *, ** and *** indicate statistical significance at the 10%, 5% and 1% level, respectively.

Table S14: Performance of Students in Classrooms with Top-Performing Girls and Boys

	(1)	(2)	(3)	(4)	(5)	(6)
	Top-Performing Girl		Top-Performing Boy		Difference (1) - (3)	
	Mean	SD	Mean	SD	Mean	SE
Average School Performance	14.326	2.423	14.279	2.417	0.047	0.020**
Difference Between Starting and Final Performance	0.806	0.661	0.787	0.656	0.019	0.006***

Notes: The table reports descriptive statistics of the school performance of students in grade 10. Average school performance refers to the average GPA at the end of the year on a scale 0-20. Difference between starting and final performance denotes the difference in the GPA in semester 2 and 1. Columns (1) and (2) present the results for students in classrooms with top-performing girls, whereas columns (3) and (4) present the results for students in classrooms with top-performing boys. Top performers are excluded from the analysis. Significance stars denote the results from two sample mean comparison t-tests; *, ** and *** indicate statistical significance at the 10%, 5% and 1% level, respectively.

Table S15: Mediation Analysis

	(1) STEM Performance	(2) Non-STEM Performance	(3) Conduct	(4) Track Choice	(5) College Major Choice	(6) Occupational Choice
Panel A: *Mediator: Confidence*						
Indirect Effect						
Shown Girl	0.031	0.028	0.034	0.026	0.027	0.022
	(0.026)	(0.024)	(0.029)	(0.022)	(0.022)	(0.019)
Direct Effect						
Shown Girl	0.013	0.492***	0.296***	0.179**	0.261***	0.221***
	(0.072)	(0.070)	(0.071)	(0.074)	(0.073)	(0.075)
Total Effect						
Shown Girl	0.043	0.521***	0.330***	0.206***	0.288***	0.243***
	(0.077)	(0.074)	(0.077)	(0.077)	(0.076)	(0.078)
Observations	578	578	578	578	578	578
Panel B: *Mediator: Autonomy*						
Indirect Effect						
Shown Girl	0.162***	0.159***	0.181***	0.140***	0.142***	0.111***
	(0.044)	(0.042)	(0.046)	(0.038)	(0.038)	(0.032)
Direct Effect						
Shown Girl	-0.102	0.376***	0.142*	0.080	0.150**	0.141*
	(0.073)	(0.072)	(0.072)	(0.075)	(0.073)	(0.075)
Total Effect						
Shown Girl	0.059	0.535***	0.322***	0.220***	0.291***	0.252***
	(0.077)	(0.074)	(0.076)	(0.077)	(0.076)	(0.077)
Observations	576	576	576	576	576	576
Panel C: *Mediator: Being an Example*						
Indirect Effect						
Shown Girl	0.066**	0.071**	0.082**	0.071**	0.074**	0.059**
	(0.028)	(0.030)	(0.034)	(0.030)	(0.031)	(0.026)
Direct Effect						
Shown Girl	-0.004	0.468***	0.263***	0.149**	0.215***	0.165**
	(0.075)	(0.074)	(0.074)	(0.074)	(0.073)	(0.075)
Total Effect						
Shown Girl	0.062	0.539***	0.345***	0.220***	0.289***	0.224***
	(0.077)	(0.074)	(0.076)	(0.078)	(0.076)	(0.078)
Observations	568	568	568	568	568	568
Shown STEM	Yes	Yes	Yes	Yes	Yes	Yes
Controls	Yes	Yes	Yes	Yes	Yes	Yes
Prefecture Fixed Effects	Yes	Yes	Yes	Yes	Yes	Yes
Month Fixed Effects	Yes	Yes	Yes	Yes	Yes	Yes

Notes: Mediator questions were presented after outcome questions in our survey instrument. The sequence of the mediator and the outcome questions can influence the results of the mediation analysis (Chaudoin, Gaines, and Livny, 2021). Outcomes and mediators are standardized to have a mean equal to zero and a standard deviation equal to one. In all specifications we control for demographics, teacher specializations, own history from school years, explicit biases, survey characteristics and state, month fixed effects. We control for indicators reflecting any missing values. Robust standard errors in parentheses; *, ** and *** indicate statistical significance at the 10%, 5% and 1% level, respectively.

Figure S1: Teacher Perceptions of Role Model Influences of Top Performers in Non-STEM

(A)

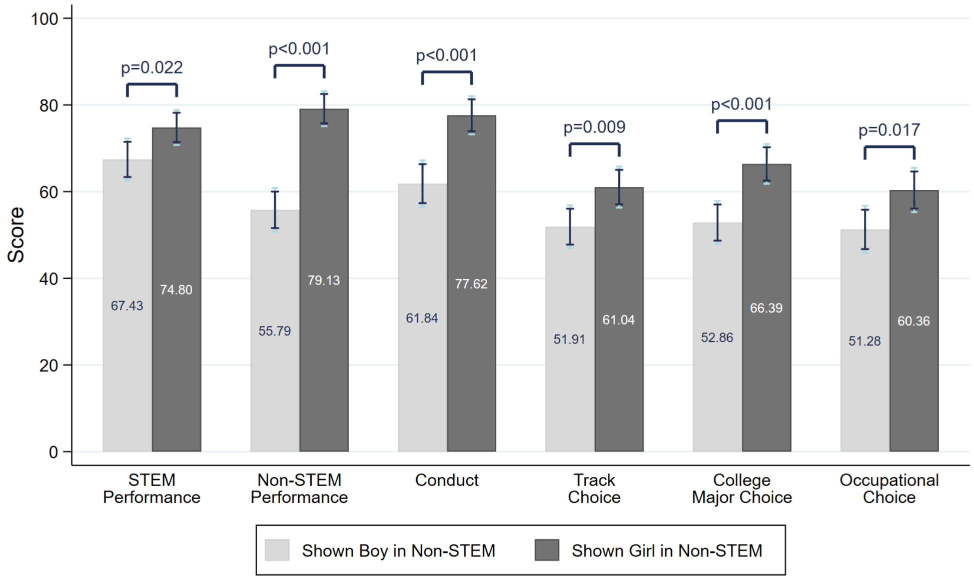

(B)

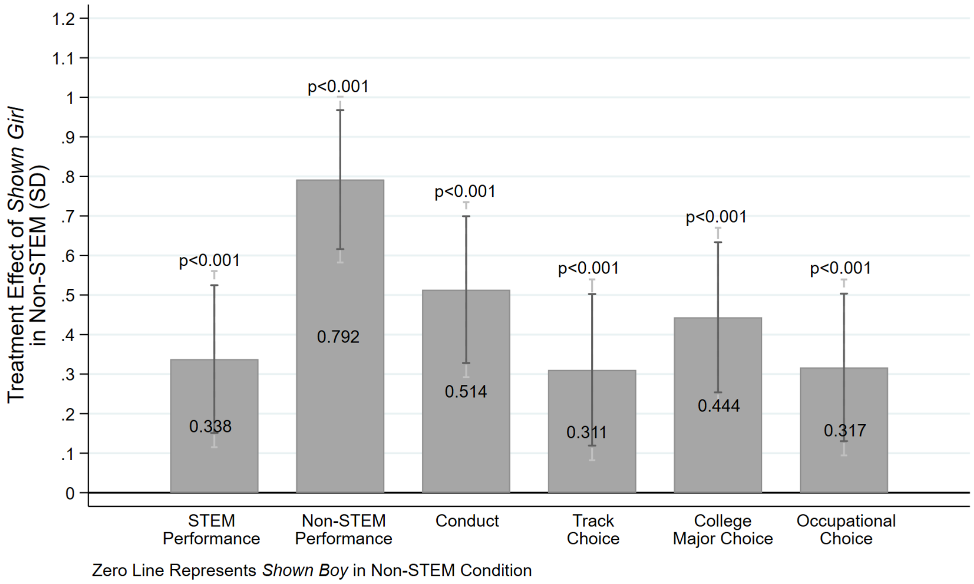

Notes: (A) presents the mean differences in teacher perceptions of role model influences of top-performing girls and boys in Non-STEM across all outcomes. The y-axis values are raw scores with a range of [0-100]. P-values denote the significance levels from two sample mean comparison tests. (B) presents the estimated difference between the treatment conditions of *Shown Girl in Non-STEM* and *Shown Boy in Non-STEM* in standard deviations, controlling for participant and survey attributes. P-values correspond to tests of statistical significance of the estimated differences. Error bars represent 90% and 95% confidence intervals.

Figure S2: Teacher Perceptions of Role Model Influences of Top Performers in STEM

(A)

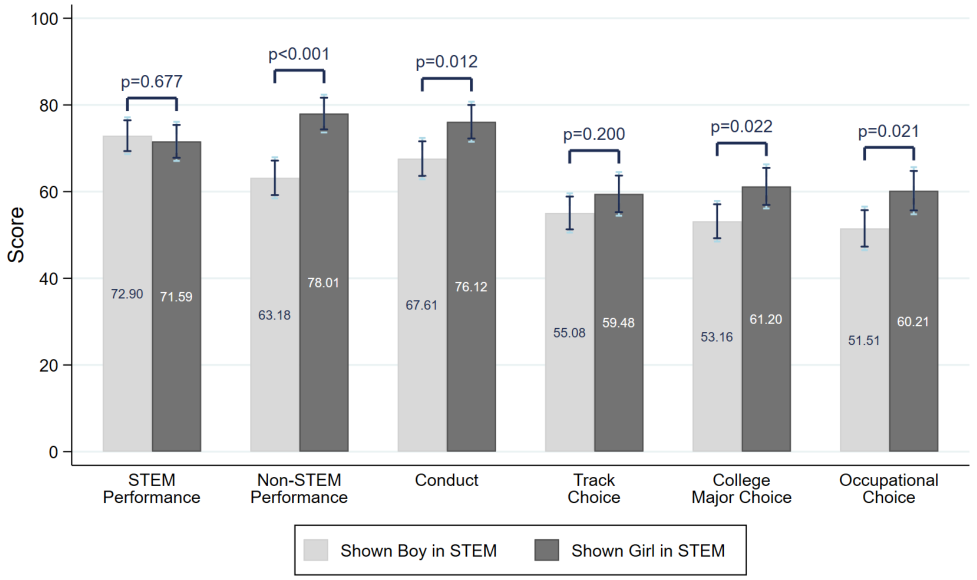

(B)

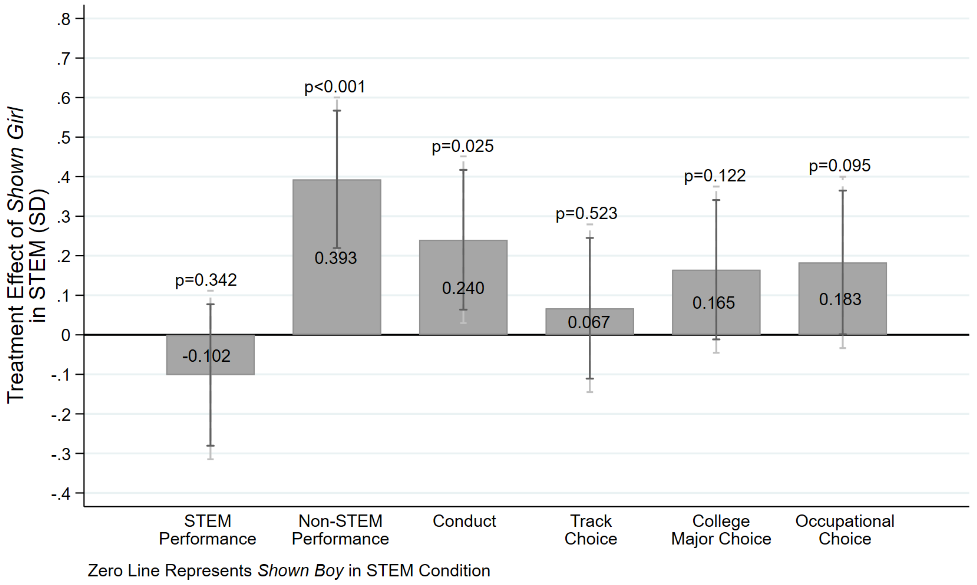

Notes: (A) presents the mean differences in teacher perceptions of role model influences of top-performing girls and boys in STEM across all outcomes. The y-axis values are raw scores with a range of [0-100]. P-values denote the significance levels from two sample mean comparison tests. (B) presents the estimated difference between the treatment conditions of *Shown Girl in STEM* and *Shown Boy in STEM* in standard deviations, controlling for participant and survey attributes. P-values correspond to tests of statistical significance of the estimated differences. Error bars represent 90% and 95% confidence intervals.

Figure S3: Perceived Role Model Qualities: Top Performers in Non-STEM

(A)

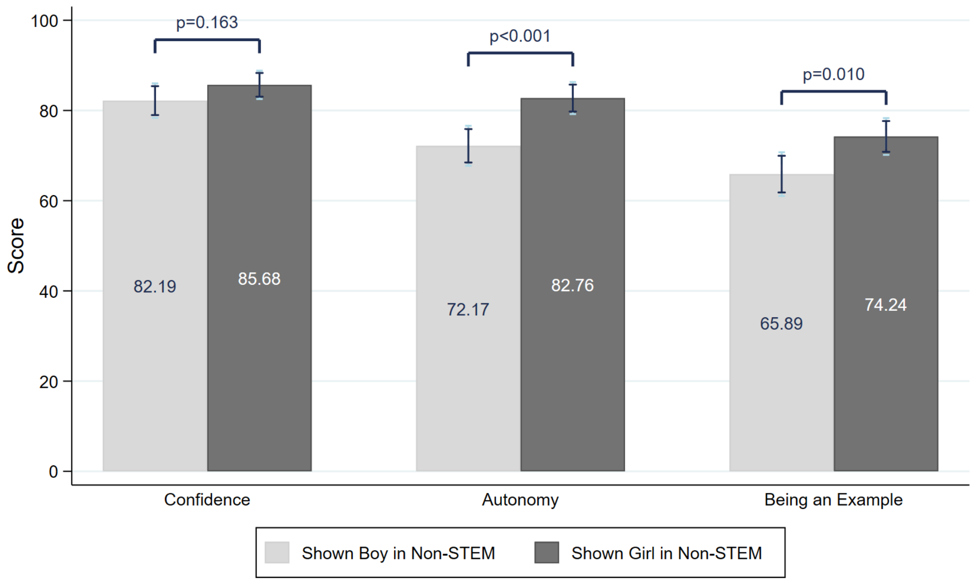

(B)

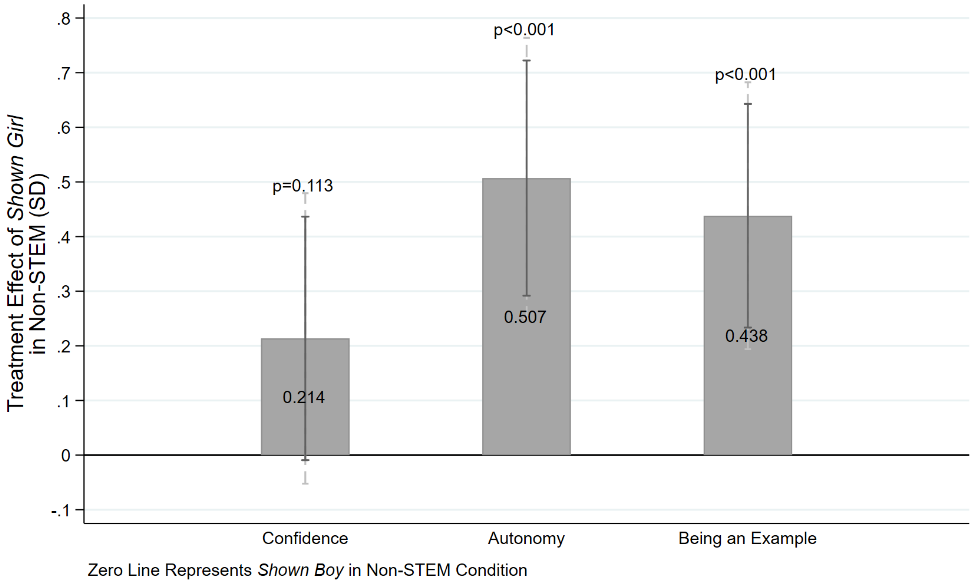

Notes: (A) presents the mean differences in the emotional conditions associated with role model influence teachers expect top-performing girls and boys in Non-STEM to experience. The y-axis values are raw scores with a range of [0-100]. P-values denote the significance levels from two sample mean comparison tests. (B) presents the estimated difference between the treatment conditions of *Shown Girl in Non-STEM* and *Shown Boy in Non-STEM* in standard deviations, controlling for participant and survey attributes. P-values correspond to tests of statistical significance of the estimated differences. Error bars represent 90% and 95% confidence intervals.

Figure S4: Perceived Role Model Qualities: Top Performers in STEM

(A)

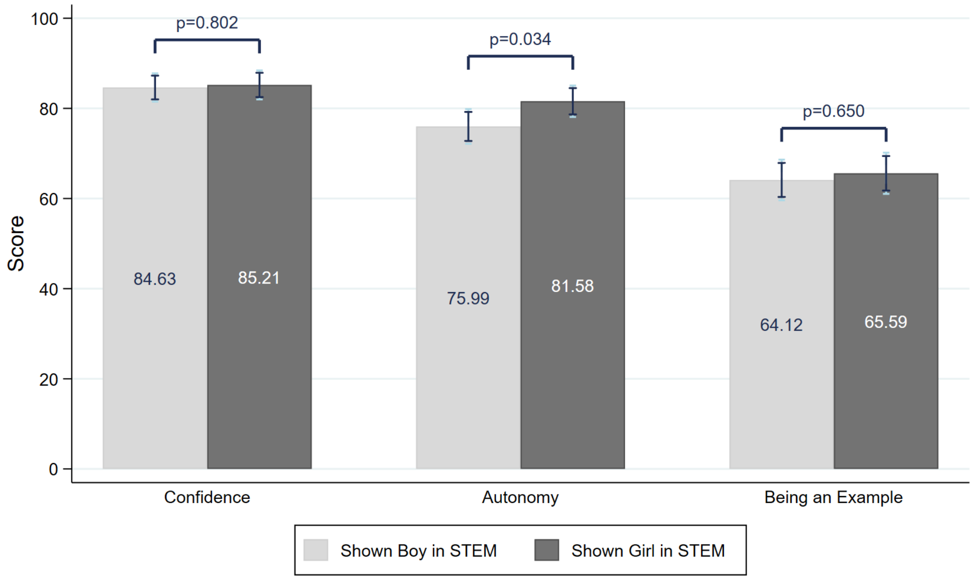

(B)

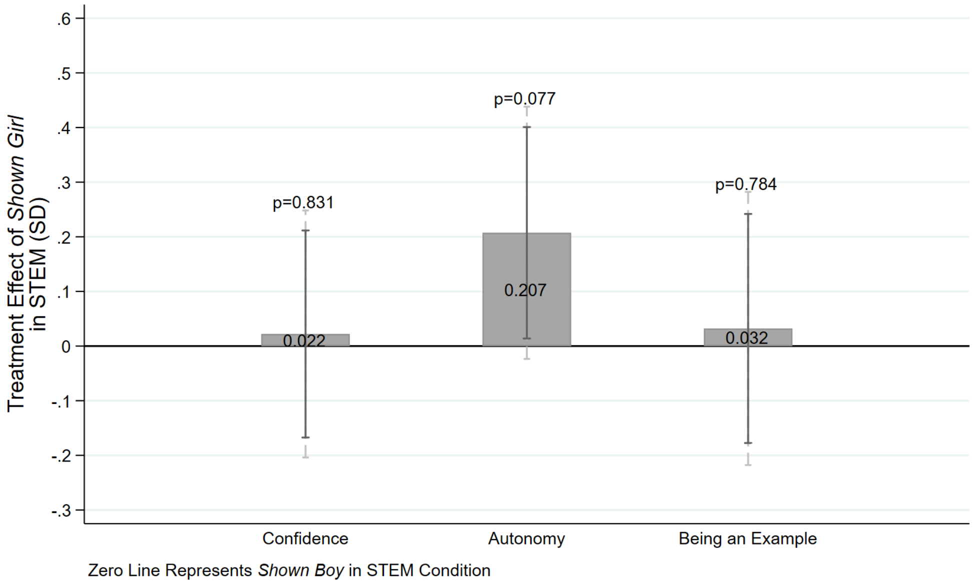

Notes: (A) presents the mean differences in the emotional conditions associated with role model influence teachers expect top-performing girls and boys in STEM to experience. The y-axis values are raw scores with a range of [0-100]. P-values denote the significance levels from two sample mean comparison tests. (B) presents the estimated difference between the treatment conditions of *Shown Girl in STEM* and *Shown Boy in STEM* in standard deviations, controlling for participant and survey attributes. P-values correspond to tests of statistical significance of the estimated differences. Error bars represent 90% and 95% confidence intervals.

Figure S5: Respondent Density

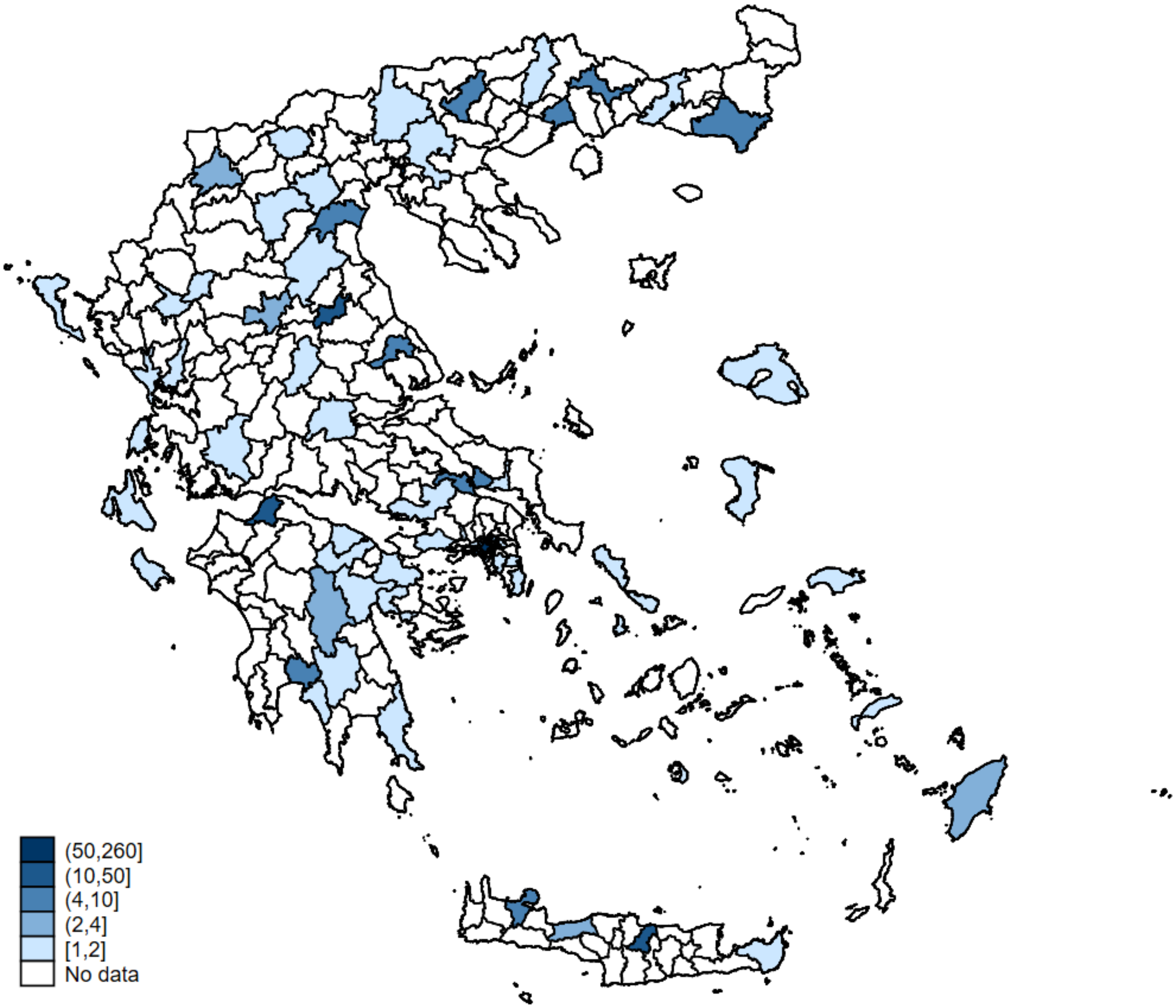

Notes: The map shows respondent density by prefecture across Greece.

Figure S6: Example of Randomized Block Question, Girl, Non-STEM

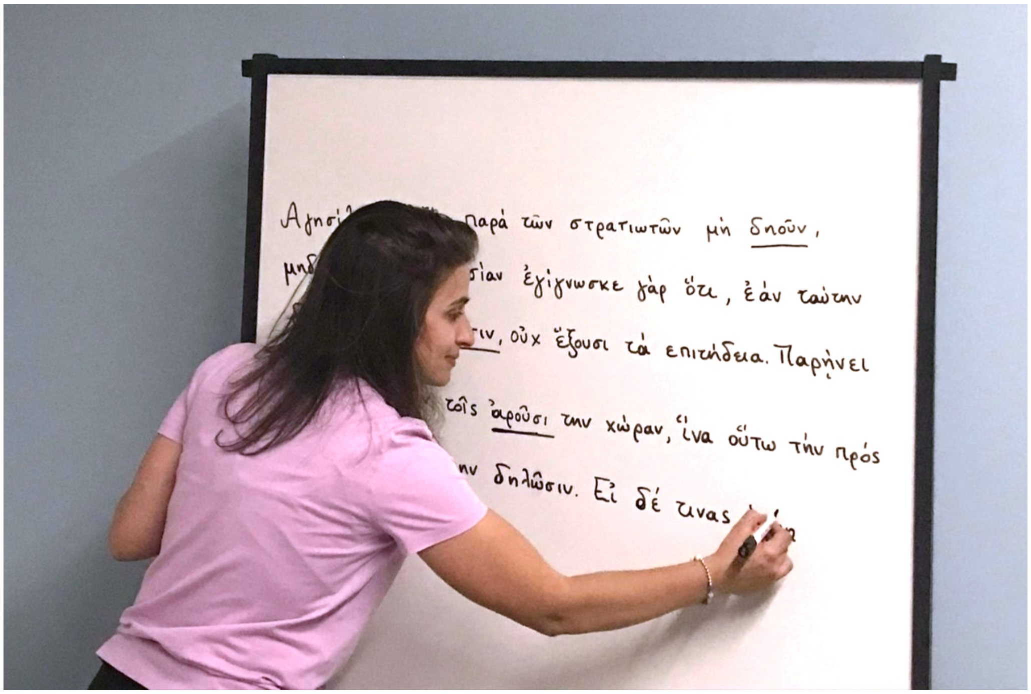

A top performing **female** student in your classroom would be impactful for others with respect to:

	**0** means Strongly Disagree and **100** means **Strongly Agree**<br>0 10 20 30 40 50 60 70 80 90 100
School performance in STEM subjects	
School performance in non-STEM subjects	
School conduct	
High school track choice	
College major choice	
Occupational Choice	

Figure S7: Example of Randomized Block Question, Girl, STEM

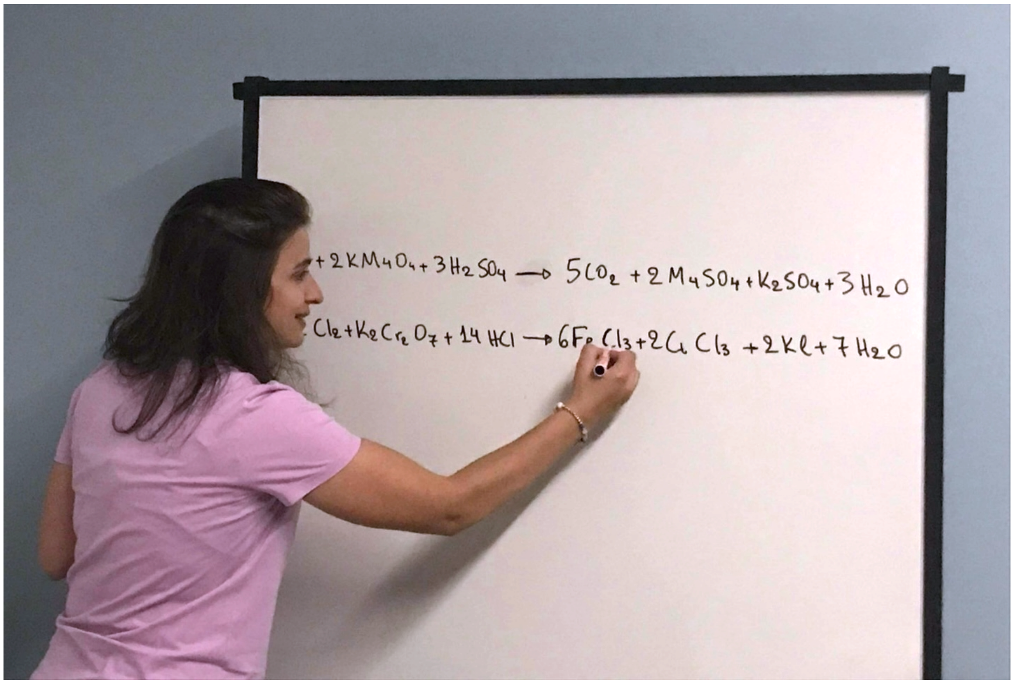

A top performing **female** student in your classroom would be impactful for others with respect to:

	**0** means Strongly Disagree and **100** means **Strongly Agree**<br>0 10 20 30 40 50 60 70 80 90 100
School performance in STEM subjects	
School performance in non-STEM subjects	
School conduct	
High school track choice	
College major choice	
Occupational Choice	

Figure S8: Example of Randomized Block Question, Boy, Non-STEM

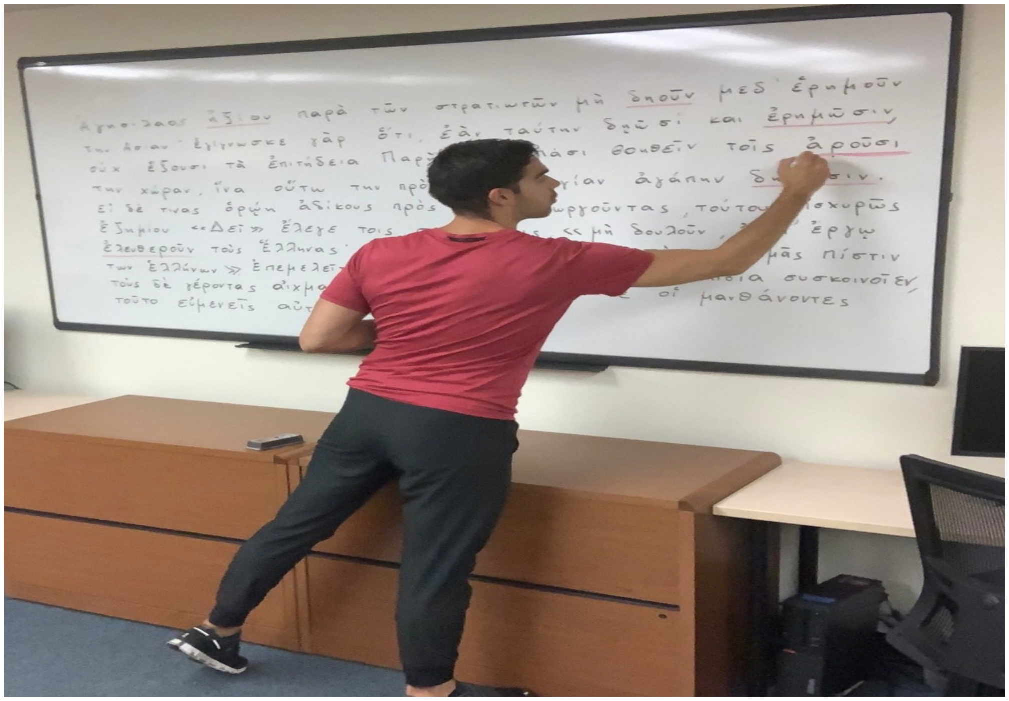

A top performing **male** student in your classroom would be impactful for others with respect to:

	**0** means Strongly Disagree and **100** means **Strongly Agree**<br>0 10 20 30 40 50 60 70 80 90 100
School performance in STEM subjects	
School performance in non-STEM subjects	
School conduct	
High school track choice	
College major choice	
Occupational Choice	

Figure S9: Example of Randomized Block Question, Boy, STEM

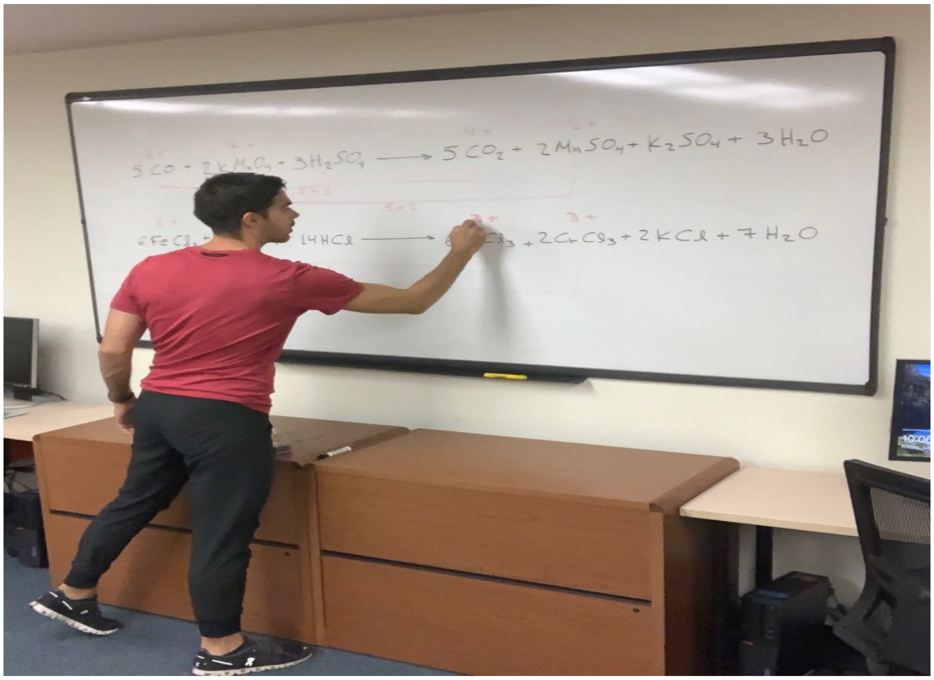

A top performing **male** student in your classroom would be impactful for others with respect to:

	**0** means Strongly Disagree and **100** means **Strongly Agree**<br>0 10 20 30 40 50 60 70 80 90 100
School performance in STEM subjects	
School performance in non-STEM subjects	
School conduct	
High school track choice	
College major choice	
Occupational Choice	

Figure S10: Survey Consent Form

Questionnaire (English)

Researchers Associate Professor Rigissa Megalokonomou and Dr. Sofoklis Goulas invite you to a study on the role model function of classmates. The following questions focus on the influences of female and male top performers during high school.

The questions refer to your recollections from high school. Participation in the survey is optional and should take no more than 9 minutes. There is no risk associated with participating or choosing not to participate, and your personal privacy is guaranteed.

Upon completing the survey, a donation of € 0.50 will be made to one of the following charitable organizations based on your selection: SOS Children's Village Greece, All Together We Can, Schedia, Kivotos you Kosmos, or another organization that you will indicate to us.

If you have any questions about the questionnaire, you can contact the researchers by email at r.megalokonomou@uq.edu.au or goulas@stanford.edu.

Should you be dissatisfied with the study's conduct, have questions, complaints, or concerns about the research or your rights as participants, please contact the Stanford Institutional Review Board (IRB) to speak with someone independent of the research organization. You can contact them by calling 650-723-2480 or mailing at Stanford IRB, Stanford University, 1705 El Camino Real, Palo Alto, CA 94306.

Under the General Data Protection Regulation (GDPR), you have certain rights regarding Your Study Data. These include the right to request access to, correct, or erase Your Study Data; object to or restrict our processing of Your Study Data; and request the transfer of Your Study Data to another organization. You may also withdraw your consent at any time. If you withdraw your consent or request Your Study Data be erased, we can still legally collect, use, and share Your Study Data up to the point in time that you withdraw your consent or request your data be erased. Even if you withdraw your consent, we may still use Your Study Data that has been anonymized or pseudonymized for specific purposes, as allowed by law. Your anonymized or pseudonymized data may be used for public health, scientific research, historical research, statistical analysis, and storage for important reasons of public interest. We will keep Your Study Data in identifiable form if required by law, and there is no limit on the length of time we will keep it for research purposes. We will also keep your Study Data to comply with legal and regulatory requirements, as long as it remains useful, unless you decide you no longer want to take part. You are allowing access to this information indefinitely as long as you do not withdraw your consent.

You consent to the collection, use, and transfer of Your Study Data, including health and other sensitive personal data, for the purpose of carrying out the research study, and know that you can withdraw your consent at any time. We will stop processing your personal data, except as described above.

Thank you for your participation.

Figure S11: All Survey Questions

==

What is your gender?
- Male
- Female
- Non-binary
- I do not wish to answer

Are you a teacher or a student?
- I am a student
- I am a primary school teacher
- I am a secondary school teacher
- I was a primary school teacher
- I was a secondary school teacher
- None of the above

[The following questions are displayed for the participants who selected "I am/was a primary/secondary school teacher" in the above question]

Do you find yourself more lenient in grading girls than boys?
- Not at all
- Little
- Probably yes
- Definitely yes

What is/was your primary subject assignment?
- PE
- …
- Other

Do you think the profession "engineer" is best suited to
- Males
- Females
- Both

Do you think the profession “lawyer” is best suited to

- Males
- Females
- Both

Do you think the profession “language teacher” is best suited to

- Males
- Females
- Both

Do you think the profession “math teacher” is best suited to

- Males
- Females
- Both

Do you have children (multiple answers)?

- Yes, I have at least a daughter
- Yes, I have at least a son
- No, I don’t have children

[RANDOMIZED BLOCKS]

[In the randomized block, participants receive a random treatment where only the questions related to the allocated treatment are displayed. Each participant receives a unique treatment.]

[Treatment 1 – A girl excelling in STEM]

[IMAGE HERE]

A top performing **female** student in your classroom would be impactful for others with respect to:

0 means Strongly Disagree and **100** means Strongly Agree

0 10 20 30 40 50 60 70 80 90 100

School performance in STEM subjects	
School performance in non-STEM subjects	
School conduct	
High school track choice	
College major choice	
Occupational choice	

Do you think the **female** student who excels in your classroom and is recognized for it feels:

0 means Strongly Disagree and **100** means Strongly Agree

	0 10 20 30 40 50 60 70 80 90 100
Greater confidence	
Greater learning autonomy	
Being an example for others	

[End Treatment 1]

[Treatment 2 – A girl excelling in Non-STEM]

[IMAGE HERE]

A top performing **female** student in your classroom would be impactful for others with respect to:

0 means Strongly Disagree and **100** means Strongly Agree

0 10 20 30 40 50 60 70 80 90 100

School performance in STEM subjects	
School performance in non-STEM subjects	
School conduct	
High school track choice	
College major choice	
Occupational choice	

Do you think the **female** student who excels in your classroom and is recognized for it feels:

0 means Strongly Disagree and **100** means Strongly Agree

	0 10 20 30 40 50 60 70 80 90 100
Greater confidence	
Greater learning autonomy	
Being an example for others	

[End Treatment 2]

[Treatment 3 – A boy excelling in STEM]

[IMAGE HERE]

A top performing **male** student in your classroom would be impactful for others with respect to:

0 means Strongly Disagree and **100** means Strongly Agree

0 10 20 30 40 50 60 70 80 90 100

School performance in STEM subjects	
School performance in non-STEM subjects	
School conduct	
High school track choice	
College major choice	
Occupational choice	

Do you think the **male** student who excels in your classroom and is recognized for it feels:

0 means Strongly Disagree and **100** means Strongly Agree

	0 10 20 30 40 50 60 70 80 90 100
Greater confidence	
Greater learning autonomy	
Being an example for others	

[End Treatment 3]

[Treatment 4 – A boy excelling in Non-STEM]

[IMAGE HERE]

A top performing **male** student in your classroom would be impactful for others with respect to:

0 means Strongly Disagree and **100** means Strongly Agree

0 10 20 30 40 50 60 70 80 90 100

School performance in STEM subjects	
School performance in non-STEM subjects	
School conduct	
High school track choice	
College major choice	
Occupational choice	

Do you think the **male** student who excels in your classroom and is recognized for it feels:

0 means Strongly Disagree and **100** means Strongly Agree

	0 10 20 30 40 50 60 70 80 90 100
Greater confidence	
Greater learning autonomy	
Being an example for others	

[End Treatment 4]

[END OF RANDOMIZED BLOCKS]

--

[The following questions are about your experiences as a student.]

In which year were you in grade 10?

____

Do you remember when you were a student, if the *top performing* student in grade 10 in your class was a girl or a boy?

- ○ Yes, they were a girl
- ○ Yes, they were a boy
- ○ No, I do not remember
- ○ I was the top performing student in my class

Do you remember when you were a student, if the *second-best* student in grade 10 in your class was a girl or a boy?

- ○ Yes, they were a girl
- ○ Yes, they were a boy
- ○ No, I do not remember
- ○ I was the second-best student in my class

If you would like to participate in our next survey, please fill in your email address below.
We will also send you the findings of our survey for your information as well as confirmation of our donations.

____

To which charity would you like us to donate the amount of money related to your completion?

- ○ SOS children’s village of Greece
- ○ Together we can
- ○ Raft
- ○ Ark of the World
- ○ Other _____

===

S1 Supplementary Survey

One may worry that any differences in the whiteboard content of boy and girl performers in the survey stimuli may affect participants' perceptions of role model influences because the whiteboard content may be perceived as indicator of hard work, effort, diligence, or attention to detail. We have conducted a supplementary survey-based experiment on the same population of our main study to test the hypothesis that the specific stimuli used could generate differential perceptions of hard work, study efforts, diligence, or attention to detail. We disseminated the survey via email during August - September 2024 targeting the same population of our main study. The sample includes 120 teachers. We were able to identify 51 teachers who also participated in our main study and use them in an additional robustness investigation of our main results that controls for their perceptions of hard work, study efforts, diligence, or attention to detail.

Table S16 in this Supplementary Appendix reports our results. If the content on the whiteboard influences participant responses, we would anticipate our survey results to favor boy actors over girl actors, particularly in Non-STEM. Our results suggest that study participants associate the girl actor (top-performing girl) with higher levels of hard work, study efforts, and attention to detail compared to the boy actor (top-performing boy) in the Non-STEM scenarios. In the STEM scenarios, the study participants associate the girl actor with higher levels of attention to detail. These results suggest that the boy actor, whose whiteboard may appear having slightly more content in the Non-STEM scenarios, is not perceived as working harder, studying more, being more diligent, or paying more attention to detail. This implies that the amount of content in the whiteboard does not influence participant responses.

Next, we replicate the baseline specification using a smaller sample derived from our supplementary survey-based experiment. In this analysis, we also control for teachers' perceptions of hard work, study efforts, diligence, and attention to detail. This allows us to assess whether the inclusion of these controls affects the effect size of our estimates. The results are presented in Table S17. Column 1 shows the replication of our baseline model using the smaller sample. Although the estimated coefficients remain consistent with the baseline results and are positive, they are not statistically significant, likely due to the reduced sample size and lower statistical power. Subsequently, we add teachers' perceptions of hard work, study efforts, diligence, and attention to detail as controls, one at a time (columns 2 to 5), and all together in column 6. In the presence of bias towards the actor who is doing more work, we would expect our estimated coefficients to decrease in magnitude with he inclusion of these controls. Our results indicate that the estimated coefficients either remain stable or slightly increase. This is in line with our previous findings, suggesting that the amount of whiteboard content does not significantly influence participant responses.

Table S16: Supplementary Survey Results

	(1) Mean	(2) SD	(3) Mean	(4) SD	(5) Mean	(6) SE
**Panel A: Overall**	Shown Girl		Shown Boy		Difference (1) - (3)	
Hard-Working	2.98	0.76	2.63	0.75	0.35	0.14**
Deligent	2.98	0.69	2.76	0.74	0.22	0.13*
Studies More Hours	2.52	0.66	2.34	0.54	0.17	0.11
Gives Attention to Details	3.04	0.72	2.49	0.72	0.54	0.13***
**Panel B: STEM**	Shown Girl in STEM		Shown Boy in STEM		Difference (1) - (3)	
Hard-Working	2.89	0.85	2.59	0.80	0.30	0.22
Deligent	2.96	0.72	2.75	0.76	0.21	0.20
Studies More Hours	2.35	0.56	2.48	0.63	-0.14	0.16
Gives Attention to Details	3.00	0.69	2.55	0.72	0.45	0.19**
**Panel C: Non-STEM**	Shown Girl in Non-STEM		Shown Boy in Non-STEM		Difference (1) - (3)	
Hard-Working	3.06	0.68	2.67	0.71	0.40	0.18**
Deligent	3.00	0.68	2.77	0.73	0.23	0.18
Studies More Hours	2.67	0.71	2.20	0.41	0.47	0.15***
Gives Attention to Details	3.07	0.75	2.43	0.73	0.64	0.19***

Notes: We investigated whether the specific stimuli used in the study are associated with differential perceptions of constructs related to hard work, study efforts, diligence, and attention to detail. The table reports descriptive statistics for teachers across all treatment groups. Panel A compares *Shown Girl* (N: 59) and *Shown Boy* (N: 61) treatment conditions. Panel B compares *Shown Girl in STEM* (N: 28) and *Shown Boy in STEM* (N: 31) treatment conditions. Panel C compares *Shown Girl in Non-STEM* (N: 31) and *Shown Boy in Non-STEM* (N: 30) treatment conditions. Significance stars denote the results from two sample mean comparison t-tests; *, ** and *** indicate statistical significance at the 10%, 5% and 1% level, respectively.

Table S17: Robustness Check: Accounting for Perceptions of Hard Work, Diligence, Study Effort, and Attention to Detail

	(1)	(2)	(3)	(4)	(5)	(6)
**Panel A: STEM Performance**						
Shown Girl	0.359	0.359	0.416	0.387	0.402	0.458
	(0.301)	(0.305)	(0.293)	(0.304)	(0.313)	(0.309)
Observations	51	51	51	51	51	51
R-squared	0.394	0.394	0.422	0.407	0.416	0.441
**Panel B: Non-STEM Performance**						
Shown Girl	0.345	0.342	0.405	0.411	0.366	0.365
	(0.367)	(0.352)	(0.358)	(0.345)	(0.384)	(0.346)
Observations	51	51	51	51	51	51
R-squared	0.210	0.277	0.242	0.281	0.215	0.324
**Panel C: Conduct**						
Shown Girl	0.087	0.084	0.171	0.160	0.137	0.152
	(0.345)	(0.334)	(0.335)	(0.307)	(0.343)	(0.328)
Observations	51	51	51	51	51	51
R-squared	0.186	0.261	0.249	0.274	0.217	0.305
**Panel D: Track Choice**						
Shown Girl	0.096	0.096	0.175	0.133	0.142	0.215
	(0.328)	(0.331)	(0.296)	(0.329)	(0.305)	(0.300)
Observations	51	51	51	51	51	51
R-squared	0.268	0.271	0.324	0.291	0.294	0.340
**Panel E: College Major Choice**						
Shown Girl	0.136	0.135	0.220	0.190	0.202	0.260
	(0.340)	(0.342)	(0.314)	(0.341)	(0.314)	(0.316)
Observations	51	51	51	51	51	51
R-squared	0.315	0.327	0.378	0.363	0.368	0.403
**Panel F: Occupational Choice**						
Shown Girl	0.259	0.260	0.300	0.261	0.266	0.322
	(0.317)	(0.322)	(0.300)	(0.316)	(0.318)	(0.326)
Observations	51	51	51	51	51	51
R-squared	0.263	0.264	0.278	0.263	0.264	0.294
Shown STEM	Yes	Yes	Yes	Yes	Yes	Yes
Controls	Yes	Yes	Yes	Yes	Yes	Yes
Additional Controls:						
Hard-Working	No	Yes	No	No	No	Yes
Diligent	No	No	Yes	No	No	Yes
Studies More Hours	No	No	No	Yes	No	Yes
Gives Attention to Details	No	No	No	No	Yes	Yes

Notes: We investigated whether the specific stimuli used in the study are associated with differential perceptions of constructs related to hard work, study effort, diligence, and attention to detail. Sample consists of 51 teachers who also participated in the initial survey. In all specifications we control for demographics, own history from school years and survey characteristics. We control for indicators reflecting any missing values. Robust standard errors are reported in parentheses.

Figure S12: Supplementary Survey Questions

==

What is your gender?

- ○ Male
- ○ Female
- ○ Non-binary
- ○ I do not wish to answer

Are you a teacher or a student?

- ○ I am a student
- ○ I am a primary school teacher
- ○ I am a secondary school teacher
- ○ I was a primary school teacher
- ○ I was a secondary school teacher
- ○ None of the above

[RANDOMIZED BLOCKS]

[In the randomized block, participants receive a random treatment where only the questions related to the allocated treatment are displayed. Each participant receives a unique treatment.]

[Treatment 1 – A girl excelling in STEM]

[IMAGE HERE]

Do you believe that the girl depicted, who excels in the classroom, works hard?
o Definitely yes
o Probably yes
o I am not sure
o Probably not
o Definitely not

Do you believe that the girl depicted, who excels in the classroom, is diligent?
o Definitely yes
o Probably yes
o I am not sure
o Probably not
o Definitely not

Do you believe that the girl depicted, who excels in the classroom, studies for many hours?
o Definitely yes
o Probably yes
o I am not sure
o Probably not
o Definitely not
Do you believe that the girl depicted, who excels in the classroom, pays attention to the details of her assignments?
o Definitely yes
o Probably yes
o I am not sure
o Probably not
o Definitely not

[End Treatment 1]

[Treatment 2 – A girl excelling in Non-STEM]

[IMAGE HERE]

Do you believe that the girl depicted, who excels in the classroom, works hard?
o Definitely yes
o Probably yes
o I am not sure
o Probably not
o Definitely not

Do you believe that the girl depicted, who excels in the classroom, is diligent?
o Definitely yes
o Probably yes
o I am not sure
o Probably not
o Definitely not

Do you believe that the girl depicted, who excels in the classroom, studies for many hours?
o Definitely yes
o Probably yes
o I am not sure
o Probably not
o Definitely not

Do you believe that the girl depicted, who excels in the classroom, pays attention to the details of her assignments?
o Definitely yes
o Probably yes
o I am not sure
o Probably not
o Definitely not

[End Treatment 2]

[Treatment 3 – A boy excelling in STEM]

[IMAGE HERE]

Do you believe that the boy depicted, who excels in the classroom, works hard?
o Definitely yes
o Probably yes
o I am not sure
o Probably not
o Definitely not

Do you believe that the boy depicted, who excels in the classroom, is diligent?
o Definitely yes
o Probably yes
o I am not sure
o Probably not
o Definitely not

Do you believe that the boy depicted, who excels in the classroom, studies for many hours?
o Definitely yes
o Probably yes
o I am not sure
o Probably not
o Definitely not

Do you believe that the boy depicted, who excels in the classroom, pays attention to the details of his assignments?
o Definitely yes
o Probably yes
o I am not sure
o Probably not
o Definitely not

[End Treatment 3]

[Treatment 4 – A boy excelling in Non-STEM]

[IMAGE HERE]

Do you believe that the boy depicted, who excels in the classroom, works hard?
o Definitely yes
o Probably yes
o I am not sure
o Probably not
o Definitely not

Do you believe that the boy depicted, who excels in the classroom, is diligent?
o Definitely yes
o Probably yes
o I am not sure
o Probably not
o Definitely not

Do you believe that the boy depicted, who excels in the classroom, studies for many hours?
o Definitely yes
o Probably yes
o I am not sure
o Probably not
o Definitely not

Do you believe that the boy depicted, who excels in the classroom, pays attention to the details of his assignments?
o Definitely yes
o Probably yes
o I am not sure
o Probably not
o Definitely not

[End Treatment 4]

[END OF RANDOMIZED BLOCKS]

Thank you.
===
